# Blood-based omic profiling supports female susceptibility to tobacco smoke-induced cardiovascular diseases

**DOI:** 10.1038/srep42870

**Published:** 2017-02-22

**Authors:** Aristotelis Chatziioannou, Panagiotis Georgiadis, Dennie G. Hebels, Irene Liampa, Ioannis Valavanis, Ingvar A. Bergdahl, Anders Johansson, Domenico Palli, Marc Chadeau-Hyam, Alexandros P. Siskos, Hector Keun, Maria Botsivali, Theo M. C. M. de Kok, Almudena Espín Pérez, Jos C. S. Kleinjans, Paolo Vineis, Soterios A. Kyrtopoulos, The EnviroGenomarkers project consortium, Ralph Gottschalk, Danitsja van Leeuwen, Leen Timmermans, Benedetta Bendinelli, Rachel Kelly, Roel Vermeulen, Lutzen Portengen, Fatemeh Saberi-Hosnijeh, Beatrice Melin, Göran Hallmans, Per Lenner, Toby J. Athersuch, Manolis Kogevinas, Euripides G. Stephanou, Antonis Myridakis, Lucia Fazzo, Marco De Santis, Pietro Comba, Hannu Kiviranta, Panu Rantakokko, Riikka Airaksinen, Päivi Ruokojärvi, Mark Gilthorpe, Sarah Fleming, Thomas Fleming, Yu-Kang Tu, Bo Jonsson, Thomas Lundh, Wei J. Chen, Wen-Chung Lee, Chuhsing Kate Hsiao, Kuo-Liong Chien, Po-Hsiu Kuo, Hung Hung, Shu-Fen Liao

**Affiliations:** 1 National Hellenic Research Foundation, Institute of Biology, Medicinal Chemistry and Biotechnology, 48 Vas. Constantinou Ave., Athens 11635, Greece; 2 Maastricht University, Minderbroedersberg 4-6, 6211 LK, Maastricht, Netherlands; 3 Department of Biobank Research, and Occupational and Environmental Medicine, Department of Public Health and Clinical Medicine, Umeå University, Sweden; 4 Nutrition Research, Department of Public Health and Clinical Medicine, Umeå University, Sweden; 5 The Institute for Cancer Research and Prevention, Italy; 6 Imperial College London, MRC-HPA Centre for Environment and Health, Department of Epidemiology and Biostatistics, School of Public Health, Faculty of Medicine, Imperial College London, St Mary’s Campus, Norfolk Place, W2 1PG, UK; 7 Department of Surgery and Cancer, Faculty of Medicine, Imperial College London, South Kensington, London SW7 2AZ, UK; 8 Division of Environmental Epidemiology, Institute for Risk Assessment Sciences, Utrecht University, The Netherlands; 9 Oncology, Department of Radiation Sciences, Umeå University, Sweden; 10 Biomolecular Medicine, Department of Surgery and Cancer, Faculty of Medicine, Imperial College London, Sir Alexander Fleming Building, South Kensington, London, SW7 2AZ, UK; 11 Centre for Research in Environmental Epidemiology (CREAL), Doctor Aiguader 88, 08003 Barcelona, Spain; 12 University of Crete, Heraklion, Greece; 13 Istituto Superiore di Sanita, Rome, Italy; 14 National Institute for Health and Welfare, Kuopio, Finland; 15 University of Leeds, UK; 16 Lund University, Sweden; 17 National Taiwan University, Taipei, Taiwan

## Abstract

We recently reported that differential gene expression and DNA methylation profiles in blood leukocytes of apparently healthy smokers predicts with remarkable efficiency diseases and conditions known to be causally associated with smoking, suggesting that blood-based omic profiling of human populations may be useful for linking environmental exposures to potential health effects. Here we report on the sex-specific effects of tobacco smoking on transcriptomic and epigenetic features derived from genome-wide profiling in white blood cells, identifying 26 expression probes and 92 CpG sites, almost all of which are affected only in female smokers. Strikingly, these features relate to numerous genes with a key role in the pathogenesis of cardiovascular disease, especially thrombin signaling, including the thrombin receptors on platelets F2R (coagulation factor II (thrombin) receptor; PAR1) and GP5 (glycoprotein 5), as well as HMOX1 (haem oxygenase 1) and BCL2L1 (BCL2-like 1) which are involved in protection against oxidative stress and apoptosis, respectively. These results are in concordance with epidemiological evidence of higher female susceptibility to tobacco-induced cardiovascular disease and underline the potential of blood-based omic profiling in hazard and risk assessment.

Exposure to tobacco smoke is one of the best studied examples of an exposure with proven causal association with a large number of human diseases^1^. Although the relevant epidemiological evidence is not completely consistent, many studies have provided evidence of differential sex susceptibility to the health effects of tobacco, especially in relation to smoking-induced cardiovascular disease (CVD; including acute myocardial infarction and coronary heart disease)^2^,^3^,^4^, chronic obstructive pulmonary disease (COPD)^1^,^5^,^6^ and lung cancer^7^,^8^,^9^. Focusing in particular on CVD, a systematic review and meta-analysis of data from 86 prospective studies and nearly 4 million subjects came to the conclusion that female smokers have a 25% higher risk of developing coronary heart disease than males with the same exposure to tobacco smoke and after allowing for other risk factors^2^, a conclusion supported by the results of a recent meta-analysis of the available data^3^. Another recent systematic review and meta-analysis covering data from 81 cohorts and nearly 4 million subjects concluded that the risk of stroke in Western populations is 10% higher in female smokers^4^.

Biomarker-based investigations have contributed significantly to our understanding of the disease risks associated with exposure to environmental hazards. Currently such biomarker studies are benefiting from the expanding use of genome-wide profiling (omics). We have recently reported the results of a study of the impact of tobacco smoke exposure on genome-wide gene expression and DNA methylation in white blood cells (WBCs) of apparently healthy smokers^10^ and identified large numbers of transcripts and DNA CpG sites whose expression and methylation, respectively, differ significantly between current and never smokers. Furthermore we used disease connectivity analysis to show that the corresponding gene profiles can identify with remarkable efficiency (specificity 94%, positive predictive value 86%) most diseases and conditions independently known to be causally associated with smoking^10^. In view of this finding, we decided to look for sex-related differences in these profiles which might possibly reflect differential disease susceptibility.

## Results

In order to minimise statistical power problems, we focused our search for sex-related differences in the effects of tobacco smoking on the transcriptomic and epigenetic features which we previously found to differ significantly between mixed-sex groups of current and never smokers^10^. These features consist of 1,273 CpG sites (FDR < 0.05; associated with 725 differentially methylated genes - DMGs) and 350 transcripts (FDR < 0.10; associated with 271 differentially expressed genes - DEGs) which were derived from the comparison of genome-wide transcriptomic and epigenetic profiles of 143 current and 311 never smokers (including 134 males and 320 females) derived from 2 cohorts, the Northern Sweden Health and Disease Study and EPIC Italy (Suppl. Tables S1 and S2).

**Figure 1:**
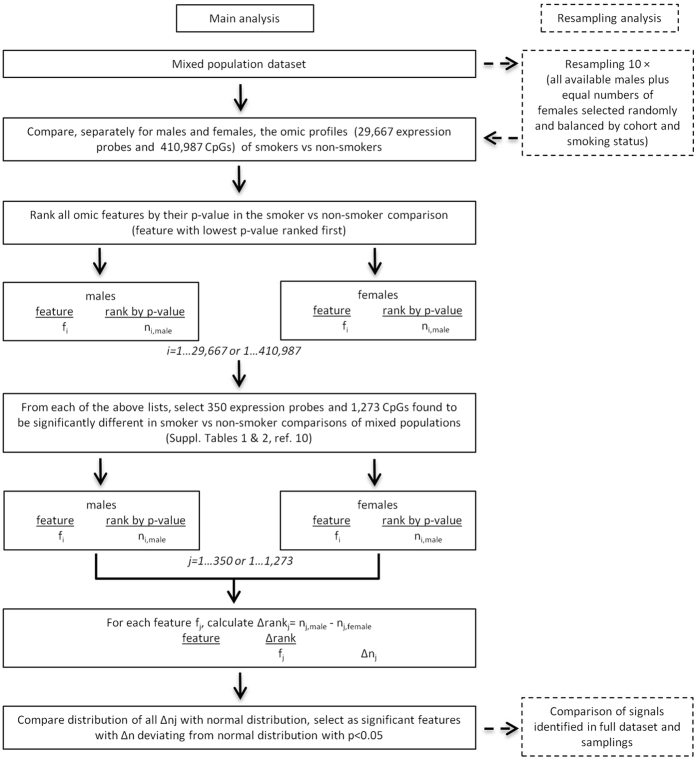
Workflow of the statistical analysis.

The tobacco exposure data available to us (Table 1) included the number of cigarettes smoked per day and smoking duration in both cohorts, the smoking intensity measured in pack-years (only in the Italian cohort), as well as plasma cotinine levels for a fraction of the study subjects from both cohorts. Inspection of this data did not reveal statistically significant differences between the two sexes, although it did suggest possibly higher exposure intensity (pack-years) in males (Table 1). While the above parameters provide an approximate picture of smoking exposure, they do not allow an accurate quantitative estimation of the long-term exposure to tobacco smoke of the different subjects suitable for use in statistical adjustment for the purpose of sub-group comparison. For this reason, and also having in mind our previous observation of the highly skewed distribution of the expression or methylation differences between current and never smokers (effect size), we opted to base our sex comparisons not on the effect size, which is expected to be strongly dependent on exposure level and duration, but on the ranking of the various features by statistical significance (i.e. p-values in current vs never smoker comparisons) in sex-stratified analyses. Thus we compared the full set of signals separately for males and females, ranking them according to the statistical significance of their current-versus-never smoker differences in each sex and, finally, compared the rankings in the two sexes of the limited number of signals of interest. The methodology is described in detail in Methods while the workflow of the procedure is shown diagrammatically in Fig. 1. We adopted this approach on the reasonable assumption that any differences between the sexes with regard to the level or duration of exposure would in general affect the effect size for the different features in a similar manner but would be unlikely to alter their ranking within each sex group. This non-parametric approach has the added advantage that it minimizes the impact of non-normal data distribution, outliers and differences in statistical power arising from the different sizes of the male and female populations.

Suppl. Fig. S1 shows the distribution of the p-values of the rank differences in the two sexes. It can be seen that a biomodal distribution is observed, with the anticipated pattern of decreasing numbers of signals as p-values decrease being observed along with an increased number of signals with very low p-values (<0.05) increases. The latter indicates the existence, among both the transcriptomic and the epigenetic signals, of sub-groups of signals whose ranking differences between the sexes are more significant than expected.

Tables 2 and 3 show the lists of expression probes and CpG sites, respectively, whose significance rankings differ significantly (p < 0.05) between the two sexes. There was no significant difference in the corresponding rankings of these signals between male and female never smokers (results not shown), indicating that the observed sex-related differences reflect the differential impact of tobacco smoke exposure.

### Expression profile

All 26 expression probes in Table 2 show large ranking differences between the sexes, with 23 exhibiting higher significance in females (median ranks: 111 in females and 19,775 in males), while the remaining 3 probes show higher significance in males. All probes are underexpressed in smokers with the exception of 2 which are overexpressed particularly in male smokers. Owing to population size limitations, many of the identified signals do not reach statistical significance in the sex-stratified analyses. However, for the 23 female-specific probes, the median FDR value with regard to the current-versus-never smoker comparisons is 0.40 (range 0.01–0.91) in females while all 23 have FDR > 0.80 in males (not shown), suggesting that the effects of smoking are largely limited to females. Importantly, as shown in Table 2 these significance ranking differences are accompanied by a corresponding difference in effect sizes for all the signals identified, with the median effect size (ratio of expression in current divided by never smokers) observed being 0.80 (range 0.72–0.93) and 0.96 (range 0.93–1.00) in females and males, respectively. No expression probe shows opposite effects of smoking in the two sexes.

### Epigenetic profile

Turning to the epigenetic profile, the 92 CpG sites (associated with 72 genes) thus identified (Table 3) show both under- and overmethylation in smokers while their ranking differences between the sexes are also substantial, with the median rank values being 432 and 215,917 in females and males, respectively. The median FDR value of these CpG sites is 0.12 (range 0.0002–0.68) in females, while all 92 sites have FDR > 0.9 in males. In complete analogy to what is observed for the transcriptomic profile, for all CpG sites the impact of smoking (Δβ = β_smokers_ − β_non-smokers_) is greater in females than in males, with the median absolute Δβ values being 1.55% (range 0.15–8.45%) and 0.33% (range 0.00–8.45%), in females and males, respectively. No CpG sites exhibit opposite effects of smoking in the two sexes. There was no statistically significant overabundance in the distribution of the CpG sites in relation to their locations (TSS200, TSS1500, body, 3′UTR, 5′UTR, 1st exon, intragenic) or to their occurrence in CpG islands and their regions (island, non-island S-shore, S-shelf, N-shore, N-shelf). Finally, we note that one gene, PLIN5, appears to be more overexpressed in females but more undermethylated in males, implying a possibility of differential epigenetic regulation in the two sexes.

### Consistency and stability of observed sex effects

Owing to the limited population size no direct replication between the two cohorts was conducted. However, cohort-stratified analyses show that the features listed in Tables 3 and 4 tend to be among the most significant features, in terms of sex differences, also in the individual cohorts. Thus, the median rank value of the 92 CpG sites of Table 4 was 3,048 (out of a total of 410,987 sites examined; top 0.7%) in females and 195,213 in males in NSHDS, and 9,098 (top 2%) and 218,704, respectively, in EPIC Italy, thus demonstrating a clear trend of higher female sensitivity in both cohorts. A similar trend was seen with the corresponding transcriptomic signals of Table 2, with the 23 female-specific signals having median ranks of 585 (out of a total of 29,667 probes examined; top 2%) in females and 25,742 in males in NSHDS, and 1,708 (top 6%) and 12,338 in females and males, respectively, in EPIC Italy. As regards the 3 male-specific transcriptomic signals, all 3 in NSHDS and 2 out of 3 in EPIC ranked higher in males.

The observation that almost all sex-specific features identified show higher sensitivity in females is striking. For comparison it is noted that only 116 of the 350 transcripts and 1,009 of the 1,273 CpGs differentially modified by smoking in the mixed population have lower FDR values in females. Although, as stated above, the rank-based comparison we employed is not expected to be significantly affected by group size, in view of the larger number of females in our study (320 female versus 134 male current and never smokers), we ran the same analysis as described above 10 times, in each case using all 134 male subjects and an equal number of females sampled randomly from our population while maintaining constant the proportions of subjects coming from each of the two cohorts and with the different types of smoking status (see Fig. 1, right). In each such resampling analysis, the resulting sex-specific epigenetic or transcriptomic signals included on average 50.3% (S.D. 7.3%) and 50.8% (S.D. 14.2%) of the signals shown in Tables 2 and 3, respectively, while the cumulative % overlap (average of each successive resampling round plus all preceding ones) for both lists tended towards approx. 50%, reflecting the similar loss of statistical power of the smaller subpopulations employed (Suppl. Table S3 and Suppl. Fig. S2). These observations provide confirmation that the identification of the sex-specific signals was not subject to bias by the group size of each sex class.

## Discussion

A number of recent studies on smoking-induced changes in methylation profiles, which employed effect size as the response classification parameter, failed to detect any sex-specific responses^11^,^12^, possibly because of residual confounding arising from insufficiently accurate adjustment for tobacco smoke exposure. By comparing in sex-stratified analyses the significance ranking of transcriptomic and epigenetic signals previously shown to differ between current and never smokers in a mixed-sex population, we identified a number of features which exhibit significantly different responses in the two sexes. Because the highly stringent nature of our non-parametric, rank-based statistical methodology inevitably attenuates sensitivity, it is possible that additional features with sex-specific behaviour may exist. On the other hand this approach has the advantage that it minimizes false positive findings and maximizes specificity.

Our observation that almost all features identified show stronger responses in females implies the possibility of higher female susceptibility to diseases related to the corresponding genes. The most notable observation regarding the list of sex-specific DEGs and DMGs relates to the presence of multiple genes related to CVD, especially genes involved in thrombin signaling and vascular and endothelial cell function. Table 4 summarises the relevant evidence (discussed further below) and compares the changes observed in the present study with those reported in clinical studies, where such information is available. It can be seen that the direction of change reported in these studies is in concordance with that which we have observed in apparently healthy female smokers, supporting the relevance of these changes to disease pathogenesis.

### Genes involved in thrombin signaling

Thrombin, a serine protease, has an essential role in coagulation and haemostasis mediated by platelets, while in addition it elicits important effects in endothelial and vascular smooth muscle cells (VSMC). For these reasons thrombin-mediated effects are of great importance in the pathogenesis of CVD. Most of the cellular effects of thrombin are initiated via the activation of a family of G-protein-coupled receptors called protease-activated receptors (PARs), which are transmembrane proteins expressed on different types of cells including platelets, endothelial cells and VSMC. The main thrombin receptor on platelets and blood vessel cells is PAR1, also known as coagulation factor II (thrombin) receptor (F2R). This gene plays a key role in vascular function and CVD^13^ and its genetic variants are known to influence platelet function^14^,^15^. In our previously published analysis of the effects of smoking on transcriptomic and epigenetic profiles^10^ we found F2R to be differentially underexpressed in current smokers with a statistical significance of FDR = 0.15 which just fails to reach the threshold adopted in the present analysis (FDR < 0.10). Inclusion of the F2R-related expression probe together with the 350 probes with FDR < 0.10 in the sex-stratified analysis described above reveals a highly significant female specificity of the effect of smoking on this gene. Thus, in male- and female-stratified analyses, respectively, the significance rankings for current-versus-never-smoker comparisons were 29,197 and 1,283 (p = 1.08 × 10^−4^), the FDR values 0.98 and 0.040 and the effect sizes 1.00 and 0.88.

**Figure 2:**
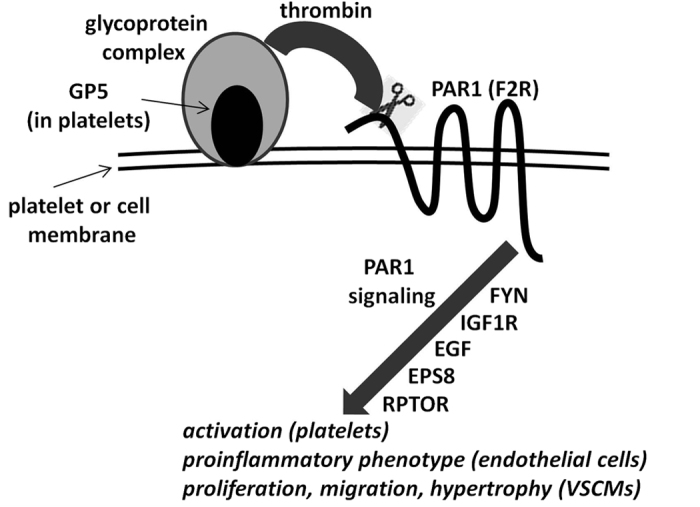
Schematic representation of some of the interactions involved in PAR1 signalling initiated by thrombin. All genes shown, are preferentially modified in female smokers, PAR1 being differentially expressed and the others differentially methylated. The genes shown near the arrow participate in signalling downstream of PAR1 and the order in which they are shown does not imply a sequential interaction.

The interaction of thrombin with PAR1 in platelets is facilitated by its initial binding to the GPIb-IX-V complex which plays a critical role in thrombosis, atherogenesis and inflammation^16^. This complex includes the glycoprotein GP5, which is associated with a CpG site we found to be differentially overmethylated in female smokers (Table 3). Following its activation via the cleavage of its N-terminal domain by thrombin, PAR1 initiates multiple kinase signaling pathways which lead to different effects depending on the cells concerned (Fig. 2). Such effects include hemostasis and thrombosis in the case of platelets, induction of pro-inflammatory phenotype in the case of endothelial cells, increase of vascular permeability, proliferation, migration and hypertrophy in the case of VSMCs, thus contributing to the pathogenesis of different types of CVD.

Signaling by the activated PAR1 receptor is controlled by, among other factors, Src kinases^17^, including FYN (FYN proto-oncogene, Src family tyrosine kinase) which is associated with a CpG site differentially undermethylated in female smokers. Following initiation of GPIb-IX-V/PAR1 signalling, FYN phosphorylates PKCδ (protein kinase Cδ) which subsequently negatively modulates platelet activation^18^. The importance of FYN for haemostasis-related disease is underlined by the report that FYN-deficient mice show an altered haemostatic response^19^.

Another gene which influences platelet function is IGF1R (insulin-like growth factor 1 receptor), which is associated with a CpG site differentially overmethylated in female smokers. The IFG1R protein is expressed at high levels on the plasma membrane of platelets while its ligand, IGF1, is a growth factor found in the α granules in platelets. Stimulation of platelets with IGF1 results in rapid phosphorylation of IGF1R and potentiation of PAR1-induced platelet aggregation^20^.

As already mentioned, thrombin-mediated PAR (including PAR1) signaling also operates in VSMC and endothelial cells, thereby playing an important role in diverse cellular activities related to inflammation, CVD, tumor growth and other conditions. In this context the genes discussed above (with the exception of GP5 which is expressed only in platelets) can be anticipated to affect by analogous mechanisms the pathogenesis of such diseases. Moreover, a number of additional genes of relevance to thrombin signaling in VSMC and endothelial cells is included in the list of genes found to be differentially modified in female smokers. One of these genes is EGF (epidermal growth factor), differentially overmethylated in female smokers, a potent mitogenic factor in many cell types acting through its receptor EGFR. Activation of EGFR promotes thrombin-induced proliferation of VSMC^21^, while its inhibition attenuates thrombin-stimulated signalling along the PI3K-Akt-mTOR-S6K1 axis, leading to effects on cell proliferation and motility^22^. Importantly, EGFR signalling is coordinated by EPS8 (epidermal growth factor receptor pathway substrate 8)^23^, which is underexpressed in female smokers. In support of a probable role of EPS8 in vascular disease is the report that EPS8-null mice show increased vascular permeability^24^. Finally, RPTOR (regulatory associated protein of MTOR, complex 1), overmethylated at 2 CpG sites in female smokers, negatively regulates mTOR kinase^25^ which, as mentioned above, is involved in thrombin signaling in VSMC. It is noted that mice with an RPTOR deletion targeted on the myocardium have been reported to develop dilated cardiomyopathy^26^.

### Other genes related to CVD pathogenesis

The list of genes exhibiting sex-specific response to tobacco smoking includes a number of additional members for which there is significant clinical or mechanistic evidence, including evidence from transgenic animal studies, that they are linked with CVD pathogenesis. The gene with the largest expression change in female smokers is HMOX1 (haem oxygenase 1), well known for its antioxidant and anti-inflammatory properties as well as for its protective role against CVD^27^,^28^,^29^. HMOX1 is also known to protect against smoking-induced COPD^30^, a disease for which there is strong evidence of differentially higher susceptibility in female smokers^1^,^5^,^6^. Another gene of interest is HDAC4 (histone deacetylase 4), differentially overmethylated in female smokers, which plays a global role in the epigenetic control of gene expression by modifying histones as well as non-histone proteins^31^ and plays an important role in regulating hypertrophic responses^32^. Finally of particular note is the female-specific demethylation of the well known anti-apoptotic gene BCL2L1 (BCL2-like 1), which plays a key role in the regulation of platelet activation and apoptosis^33^. Among the consequences of platelet apoptosis is the production of microparticles, which are recognised to play an important role in inflammation, CVD, coagulation and angiogenesis^34^.

The largest sex-related difference in the impact of smoking (absΔΔβ, last column in Table 4) is observed at 3 CpG sites associated with CACNA1D (calcium channel, voltage-dependent, L type, alpha 1D subunit). This gene encodes for the cav1.3 subunit of a voltage-gated, L-type calcium channel and human and animal studies strongly support its association with various pathological conditions, including cardiovascular and neurological disorders^35^,^36^,^37^,^38^. On a side note, it is of interest that cav1.3 physically interacts with the receptor of GABA_B_, with activation of the latter leading to an increase in the L-type calcium channel currents^39^. Given the key role of this receptor in the mechanism of addiction, it is possible that any sex-related variation in CACNA1D expression may be reflected in corresponding differences in susceptibility to nicotine addiction. In support of this idea, several lines of evidence indicate that females have a higher susceptibility to nicotine dependence, including faster progression to dependence, shorter and less frequent abstinence periods, greater difficulty to quit, and poorer response to smoking cessation treatments^38^,^40^,^41^.

Other genes listed in Table 4, for which there is evidence of varying strength of links with different types of CVD, include TAGLN (transgelin^42^), SYNE1 (spectrin repeat containing, nuclear envelope 1)^43^,^44^, IL32 (interleukin 32)^45^, PLIN5 (perilipin 5)^46^, HNRNPUL1 (Heterogeneous Nuclear Ribonucleoprotein U-Like 1)^47^ and miR-30d^48^. Finally, Table 4 shows a number of genes (C14orf43, C1orf21, ST3GAL1 and ZNF19) which do not have any function related to CVD pathogenesis, however they have been reported to be differentially expressed in patients with different types of CVD.

### Molecular basis of sex-specific effects of tobacco smoke exposure

The previous discussion shows that numerous genes among those found to be differentially altered in female smokers interact closely in the context of thrombin signaling in platelets and vascular/endothelial cells. While the molecular basis for such differential female susceptibility to tobacco smoke is not currently understood, there is strong evidence that thrombin signaling and hemostasis are subject to hormonal influences and it is possible that such influences may also modify the responses to tobacco smoking. Megakaryocytes and platelets express the estrogen and androgen receptors, and the coagulation cascade is known to be influenced by variations in the levels of female sex steroids^49^,^50^,^51^, while it has been suggested that females have greater baseline platelet reactivity which may be attenuated by estrogens^51^,^52^. Furthermore, it has been reported that platelets from women are more responsive than those of men to thrombin agonists^51^, and that females with atherosclerosis show higher PAR1-mediated platelet activation^53^. Sex hormones contribute to the modulation of additional genes related to CVD and may therefore also modify the impact of smoking. For example, the expression of HMOX1 under conditions of oxidative stress is modulated by estrogen receptor alpha^54^, while estrogen receptor beta modulates the expression of HDAC4 under the influence of hypertrophic factors in rat cardiomyocytes^55^.

Summarising the preceding discussion, a large number of genes which are known or suspected to play a role in the mechanism of CVD, and to modulate corresponding disease risks, have their expression or methylation in WBCs modified by smoking significantly more in females than in males. It is not known whether similar changes occur in other tissues of smokers. However we have recently reported that tobacco smoking causes similar changes in expression and CpG methylation in the Ah receptor repressor gene in WBCs and lung cells^10^. We have also shown that the genes which are differentially expressed or methylated in WBCs of smokers are closely related to many smoking-induced diseases regardless of their target tissue, implying that changes observed in blood cells may reflect more global effects. This conclusion is further supported by the concordance of the results presented in the present study with the conclusions of epidemiological studies which consistently point to a higher female susceptibility to tobacco-induced CVD. Furthermore, we also report analogous, although more limited, findings supporting higher female susceptibility to tobacco-induced COPD and nicotine addiction. Although our evaluation of the disease-relevance of the sex-specific DEGs and DMGs presented above did not include cancer, many of the signaling pathways discussed are also highly relevant to carcinogenesis^56^. It is noted that the current evidence regarding sex susceptibility to tobacco-related cancer is mixed^7^,^8^,^9^,^57^.

In conclusion, the results presented here underline the utility of blood-based omics profiling for identifying health hazards associated with environmental exposures and suggest a potential for use of such data in identification of susceptible sub-groups.

## Materials and Methods

The present report is based on data from the Envirogenomarkers project (www.envirogenomarkers.net). Envirogenomarkers is a prospective case-control study nested within the European Prospective Investigation into Cancer and Nutrition study (EPIC-ITALY) and the Northern Sweden Health and Disease Study (NSHDS)^58^,^59^, in which subjects asymptomatic at the time of enrolment provided a blood sample and information on dietary habits, lifestyle, health history etc. The EnviroGenomarkers project and its associated studies and experimental protocols were approved by the Regional Ethical Review Board of the Umea Division of Medical Research, for the Swedish cohort, and the Florence Health Unit Local Ethical Committee, for the Italian cohort, and all participants gave written informed consent. All methods were carried out in accordance with the approved guidelines.

Owing to the Envirogenomarkers project’s design, some of the participating subjects had been selected on the basis of the fact that they went on to develop breast cancer or B-cell lymphoma 2–16 years after recruitment, however for the purposes of the present study they were all treated as apparently healthy. We have previously shown that inclusion of such subjects did not significantly affect the list of smoking-modified transcriptomic and epigenetic features^10^. Anthropometric measurements and lifestyle parameters had been collected through questionnaires at recruitment (1993–1998 for EPIC-ITALY; 1990–2006 for NSHDS). Information on smoking was obtained through questionnaires and included data on duration, number of cigarettes smoked per day and (only in Italy) pack-years. In addition, for a fraction of the subjects data on plasma cotinine concentration were also available. Details of the subjects involved in the present study are shown in Table 1. Sample collection, storage and processing procedures have been described elsewhere^58^,^59^. Based on the conclusions of a previously published pilot study^60^, subjects were included in the study only if the processing of their blood samples and placement of their buffy coats in cold storage had been completed within 2 hours of collection so as to minimize effects on the transcriptomic profile.

RNA and DNA extraction from buffy coats, genome-wide analysis of gene expression (Agilent 4 × 44 K human whole genome microarray platform) and CpG methylation (Illumina Infinium HumanMethylation450 platform) and the corresponding data quality assessment and preprocessing, were conducted as described previously^60^. Cotinine levels (AUC) in plasma were measured by reverse-phase chromatography on an Acquity UPLC system (Waters Corporation, Milford, MA, USA) with a Acquity HSS T3 C18 10 mm × 2.1 mm, 1.8 μm, column (Waters) and a binary gradient elution comprising of water 0.1% formic acid and acetonitrile 0.1% formic acid for 19 min. Online analysis of the eluent was performed using a quadrupole time-of-flight mass spectrometer (QTOF-Ultima-MS; Waters) in the positive ion mode. Data were processed using Databridge and XCMS software (Waters). We confirmed the identity of cotinine with authentic standard and accurate mass.

Data analysis and the derivation of lists of expression probes and CpG sites which differed significantly between current and never smokers has been described in detail previously^10^. Briefly, linear mixed models were ran, using M values for DNA methylation or log2 intensities of mRNA expression as dependent variables, plus date of isolation, labeling, and hybridization for RNA expression, or date of analysis for methylation, as random variables. All analyses additionally adjusted for sex, age, BMI and cohort. Owing to the design of the EnviroGenomarkers project, future disease (breast cancer, B-cell lymphoma) and case-control status were also included as fixed variables. In the case of DNA methylation data, the models were also adjusted for blood cell composition estimated with a published algorithm^61^. Multiple testing was accounted for with high stringency by using Bonferroni or FDR Benjamini-Hochberg correction. This procedure led to the identification of lists, recently published^10^, of 1,273 CpG sites (FDR < 0.05) and 350 transcripts (FDR < 0.10) which differ significantly in current relative to never smokers (Suppl. Tables 1 and 2).

We looked for sex-related differences among the above mentioned expression and DNA methylation features by employing rank-based, non-parametric, statistical testing methodology based on the evaluation of the differences in the significance ranks of the probes in the two classes (Fig. 2). Towards this end we first conducted current-versus-never smoker comparisons, using the same statistical models as previously, for all transcriptomic and epigenetic features (29,667 expression probes and 410,987 methylation probes, respectively) separately in males and females, ranking all features by the corresponding significance (p-value). Subsequently we extracted from these lists the rank values of the features which we had previously found to be significant in the mixed population (350 expression probes significant at FDR < 0.10 and 1,273 CpGs significant at Bonferoni-corrected p < 0.05) and calculated their differences between the two sexes. We thus derived a distribution of differences which conforms to the normality assumption (steep unimodal) and was used as basis in order search for differences that violate the null hypothesis that only non-sex-related differences are observed, i.e. signal rankings in sex-stratified analyses are equal. The statistic was calculated from the corresponding complementary cumulative distribution function (Survival Function)^62^ which describes the probability that a variate takes a value greater than a particular number, taking as a significance threshold the value of p < 0.05. The workflow of this analysis is described diagrammatically in Fig. 1. The tool for the implementation of the statistical evaluation of sex-specific rank differences described above is publically available, under the name RIPOSTE (“Rank DrIven POpulation STatistical Evaluation”), on the Galaxy platform at http://mebioinfo.ekt.gr/galaxy, where instructions for its use are also given.

In order to check for any bias introduced by the difference in male and female population sizes on the selection of sex-specific signals, we implemented a permutation probabilistic approach (also illustrated in Fig. 1, right) by randomly resampling 10 times the full population so as to extract subpopulations even with respect to sex and smoking status, subsequently applying to all subpopulations thus selected the same analytical workflow as described above. In each subpopulation we used all available male subjects (134) and an equal number of females selected randomly while maintaining unaltered the proportions of females from the different cohorts and with different smoking status. For each resampling we counted the number of significant (p < 0.05 for rank difference) signals that came from among those obtained with the full male and female populations (shown in Tables 2 and 3).

## Additional Information

**How to cite this article**: Chatziioannou, A. *et al*. Blood-based omic profiling supports female susceptibility to tobacco smoke-induced cardiovascular diseases. *Sci. Rep.* **7**, 42870; doi: 10.1038/srep42870 (2017).

**Publisher's note:** Springer Nature remains neutral with regard to jurisdictional claims in published maps and institutional affiliations.

## Supplementary Material

Supplementary Information

## Figures and Tables

**Table 1:** Study population and sex comparison of smoking exposure-related parameters.

		Males	Females
	N	236	413
	age; mean (range)	51.0 (30.2–65.0)	52.8(29.6–74.9)
cohort	NSHDS; N	171	228
	EPIC Italy; N	65	185
	BMI; mean (range)	26.5 (19.0–39.5)	25.4(18.8–55.28)
smoking status	current smokers; N	43 (18.2%)	100 (24.2%)
	former smokers; N	102 (43.2%)	93 (22.5%)
	never smokers; N	91 (38.6%)	220 (53.3%)
duration of smoking	N	40	90
	years, mean (s.d)	31.8 (8.7)	30.6 (18.1)
	p	0.49	
pack-years*	N	14	45
	mean (S.D)	582.9 (429.0)	356.6 (266.0)
	p	0.06	
cigarettes/day	total N	42	98
	≤4, N	7 (16.7%)	24 (24.5%)
	5–15, N	17 (40.5%)	50 (51%)
	≥25, N	18 (42.9%)	24 (24.4%)
	p#	0.2	
plasma cotinine	N	40	35
	AUC; mean (s.d.)	33.7 (8.7)	28.1 (8.5)
	p	0.85	

^*^Only EPIC Italy; ^#^chi-squared test.

**Table 2:** Expression probes and DEGs ranked by significance of sex specificity.

Expression probe	Gene symbol	Gene name	Rank by p-value in sex-stratified analysis			Effect size (ratio current/never smokers) in sex-stratified analysis	
			Males	Females	p rank difference	Males	Females
A_23_P120883	NMOX1	heme oxygenase (decycling) 1	29,411	31	2.82E-05	1.00	0.77
A_23_P87013	TAGLN	transgelin	29,013	74	3.86E-05	1.00	0.80
A_24_P410453	SYNE1	spectrin repeat containing. nuclear envelope 1	28,573	120	5.42E-05	1.00	0.81
A_23_P23834	LGR6	leucine-rich repeat-containing G protein-coupled receptor 6	27,005	26	1.46E-04	1.49	0.72
A_23_P136347	EPS8	epidermal growth factor receptor pathway substrate 8	26,954	16	1.50E-04	0.99	0.75
A_24_P173754	C1orf21	chromosome 1 open reading frame 21	24,586	24	6.54E-04	0.98	0.78
A_23_P354387	MYOF	myoferlin	24,542	111	7.06E-04	0.98	0.79
A_23_P66881	RGS9	regulator of G-protein signaling 9	21,736	118	3.29E-03	0.96	0.79
A_24_P76644			20,804	121	5.24E-03	0.98	0.88
A_23_P86682	MYOF	myoferlin	19,999	56	7.46E-03	0.96	0.78
A_23_P43679	ZNF618	zinc finger protein 618	19,852	22	7.86E-03	0.96	0.84
A_23_P1962	RARRES3	retinoic acid receptor responder (tazarotene induced) 3	19,775	113	8.49E-03	0.97	0.87
A_23_P113161	C1orf21	chromosome 1 open reading frame 21	19,632	47	8.80E-03	0.95	0.75
A_24_P921823	TCF7L2	transcription factor 7-like 2 (T-cell specific. HMG-box)	18,329	7	1.53E-02	0.96	0.77
A_23_P45831	CHD1L	chromodomain helicase DNA binding protein 1-like	19,187	1,428	1.94E-02	0.98	0.93
A_23_P48175	TMEM106C	transmembrane protein 106C	18,096	410	2.00E-02	0.96	0.88
A_23_P39251	PLIN5	perilipin 5	229	17,725	2.16E-02	1.34	1.05
A_23_P200780	TGFBR3	transforming growth factor. beta receptor III	17,259	94	2.47E-02	0.94	0.80
A_23_P110791	CSF1R	colony stimulating factor 1 receptor	16,516	49	3.23E-02	0.95	0.80
A_32_P140475	KIAA1377	KIAA1377	72	16,014	3.93E-02	0.82	0.97
A_23_P213620	PPP2R2B	protein phosphatase 2. regulatory subunit B. beta	16,049	256	4.14E-02	0.94	0.86
A_32_P75141			15,969	240	4.24E-02	0.94	0.85
A_23_P385105	PLCD4	phospholipase C. delta 4	15,930	354	4.48E-02	0.94	0.86
A_24_P88763	LOXL3	lysyl oxidase-like 3	15,979	413	4.50E-02	0.95	0.89
A_24_P9883	DKFZp761E198	DKFZp761E198 protein	27	15,524	4.61E-02	1.49	1.05
A_23_P109171	BFSP1	beaded filament structural protein 1. filensin	15,892	481	4.75E-02	0.93	0.84

**Table 3:** CpG sites and DMGs ranked by significance of sex specificity.

Probe	Gene symbol	Gene name	Rank by p-value in sex-stratified analysis			Males			Females			absΔΔβ = abs(Δβ_fem_ −Δβ_m)_
			Males	Females	p rank difference	β* current smokers	β* never smokers	Δβ_m_#	β* current smokers	β* never smokers	Δβ_fem_#	
cg03378003	SETD2	SET domain containing 2	410,615	375	1.73E-08	84.23	84.24	0.00	84.92	83.59	1.33	1.33
cg05713794	GP5	glycoprotein V (platelet)	399,753	108	4.17E-08	81.42	81.44	−0.02	80.63	78.46	2.17	2.19
cg13411554	CACNA1D	calcium channel. voltage-dependent. L type. alpha 1D subunit	398,828	206	4.53E-08	29.34	29.25	0.08	34.69	28.22	6.47	6.39
cg19028369	C3orf19	chromosome 3 open reading frame 19	395,672	613	6.06E-08	4.45	4.46	−0.01	4.29	4.59	−0.29	−0.29
cg12611488	SKI	SKI proto-oncogene	394,498	63	6.38E-08	78.07	77.99	0.08	82.74	78.36	4.39	4.31
cg00436663	LOC400927		388,154	171	1.07E-07	4.89	4.88	0.01	4.58	5.04	−0.45	−0.47
cg17993335	DNMBP	dynamin binding protein	387,580	886	1.19E-07	79.14	79.20	−0.06	80.01	78.03	1.99	2.05
cg15743533	FAM110A	family with sequence similarity 110. member A	369,751	234	4.50E-07	11.46	11.50	−0.04	10.67	11.72	−1.05	−1.01
cg17098415			366,343	190	5.79E-07	1.83	1.84	−0.02	1.93	2.20	−0.27	−0.25
cg17532753	HDAC4	histone deacetylase 4	370,727	5.606	6.26E-07	67.37	67.24	0.13	67.71	66.06	1.65	1.52
cg25115829	SUZ12P	SUZ12 polycomb repressive complex 2 subunit pseudogene 1	179,095	698	7.57E-07	73.04	72.23	0.81	75.55	72.97	2.58	1.77
cg13543915			360,064	247	9.28E-07	61.82	61.91	−0.09	62.91	64.51	−1.60	−1.52
cg09257526	IL6R	interleukin 6 receptor	359,954	1.607	1.03E-06	20.44	20.52	−0.08	20.04	20.67	−0.62	−0.55
cg12689529	KIRREL3	kin of IRRE like 3 (Drosophila)	341,191	406	3.63E-06	6.84	6.91	−0.07	6.50	7.19	−0.69	−0.62
cg13379236	EGF	epidermal growth factor	332,044	256	6.73E-06	60.43	60.24	0.19	62.51	60.67	1.84	1.65
cg06459104	EPB41L3	erythrocyte membrane protein band 4.1-like 3	331,586	341	6.98E-06	34.31	34.76	−0.46	33.82	38.07	−4.25	−3.79
cg10232140	ERCC6	excision repair cross-complementation group 6	328,904	141	8.25E-06	1.54	1.50	0.05	1.78	1.32	0.46	0.41
cg27430293			152,757	2.427	8.33E-06	34.11	33.26	0.85	36.95	34.92	2.03	1.18
cg04985185	MBTPS1	membrane-bound transcription factor peptidase. site 1	321,636	229	1.34E-05	83.83	83.59	0.24	84.90	82.76	2.14	1.89
cg27171474	CDCA8	cell division cycle associated 8	329,032	404	2.05E-05	84.89	84.78	0.11	86.22	85.01	1.21	1.09
cg08202265	TAC1	tachykinin. precursor 1	314,885	272	2.09E-05	87.55	87.23	0.31	88.45	85.15	3.30	2.99
cg08528970			311,563	11.616	5.23E-05	13.11	13.34	−0.22	13.88	14.30	−0.41	−0.19
cg02003183	CDC42BPB	CDC42 binding protein kinase beta (DMPK-like)	296,555	442	6.59E-05	8.34	8.14	0.21	6.33	5.57	0.76	0.55
cg10665960	EPC2	enhancer of polycomb homolog 2	295,295	491	7.13E-05	4.71	4.66	0.05	4.57	4.90	−0.33	−0.38
cg26724967	IL32	interleukin 32	184,670	577	9.65E-05	59.76	60.34	−0.58	59.95	61.77	−1.82	−1.25
cg07411111	TPD52L2	tumor protein D52-like 2	289,518	998	1.04E-04	61.46	61.04	0.42	62.07	59.99	2.08	1.66
cg09374353	EHD1	EH-domain containing 1	284,155	321	1.36E-04	20.73	20.54	0.19	18.44	19.61	−1.18	−1.37
cg23670519			278,212	616	1.94E-04	27.86	27.27	0.59	27.09	24.35	2.74	2.15
cg05307957	ARID1A	AT rich interactive domain 1A (SWI-like)	277,497	132	1.97E-04	2.70	2.78	−0.08	2.99	3.38	−0.39	−0.31
cg20305005	SCN2A	sodium channel. voltage-gated. type II. alpha subunit	273,040	240	2.54E-04	58.37	57.96	0.42	58.65	56.27	2.38	1.96
cg13989999	BCL2L1	BCL2-like 1	268,258	3.290	3.89E-04	47.52	47.93	−0.41	48.03	49.34	−1.31	−0.90
cg06397161	SYNGR1	synaptogyrin 1	262,200	1.787	4.96E-04	42.48	42.79	−0.31	44.56	46.19	−1.63	−1.32
cg06959021	TCHP	trichoplein. keratin filament binding	255,728	173	6.39E-04	58.68	58.28	0.40	61.71	59.85	1.86	1.45
cg01911191			253,223	1.609	7.82E-04	2.70	2.62	0.08	2.34	2.07	0.27	0.19
cg25446789	DTNB	dystrobrevin. beta	150,180	257	8.25E-04	40.28	40.87	−0.60	40.07	42.21	−2.14	−1.55
cg14179401			249,065	983	9.35E-04	33.38	33.60	−0.22	34.75	35.82	−1.07	−0.85
cg08601457	FYN	FYN proto-oncogene. Src family tyrosine kinase	242,282	422	1.27E-03	7.11	7.28	−0.17	5.98	6.68	−0.70	−0.53
cg16702313	C14orf43	chromosome 14 open reading frame 43	239,378	153	1.45E-03	60.71	61.00	−0.29	61.85	63.45	−1.60	−1.31
cg13832372	LHX6	LIM homeobox 6	239,227	188	1.46E-03	10.96	11.42	−0.45	10.32	12.60	−2.28	−1.83
cg24242519	FAM49A	family with sequence similarity 49. member A	236,302	1.218	1.77E-03	13.47	13.75	−0.28	13.51	14.61	−1.10	−0.82
cg22638542	SEC22C	SEC22 homolog C. vesicle trafficking protein	227,163	329	2.60E-03	80.69	80.38	0.31	82.48	81.37	1.11	0.80
cg01904243	C14orf43	chromosome 14 open reading frame 43	225,047	164	2.84E-03	13.97	14.22	−0.25	12.62	13.82	−1.20	−0.95
cg06338710	GFI1	growth factor independent 1 transcription repressor	224,461	205	2.92E-03	76.24	76.90	−0.66	77.50	79.53	−2.03	−1.38
cg19048950	LOC100188		219,307	463	3.72E-03	76.17	75.70	0.46	73.42	71.15	2.28	1.81
cg02104700	S100P	S100 calcium binding protein P	217,731	496	4.00E-03	2.42	2.49	−0.07	2.45	2.67	−0.22	−0.15
cg15417641	CACNA1D	calcium channel. voltage-dependent. L type. alpha 1D subunit	215,584	77	4.31E-03	50.18	48.86	1.32	53.39	46.64	6.76	5.44
cg11222173	RPTOR	regulatory associated protein of MTOR. complex 1	216,169	1.025	4.38E-03	62.03	61.49	0.54	61.00	58.74	2.26	1.71
cg08062087	C2orf66	chromosome 2 open reading frame 66	215,666	587	4.39E-03	69.15	68.61	0.54	70.09	68.06	2.03	1.49
cg14950321	PLIN5	perilipin 5	215,623	763	4.44E-03	39.87	40.42	−0.55	41.31	43.13	−1.82	−1.27
cg08778287	IGF1R	insulin-like growth factor 1 receptor	214,383	476	4.62E-03	33.41	32.78	0.64	31.37	28.84	2.54	1.90
cg13698937	C4orf46	chromosome 4 open reading frame 46	210,788	694	5.45E-03	76.64	75.97	0.67	77.47	75.32	2.15	1.49
cg00357551	FAM196B	family with sequence similarity 196. member B	207,522	1.016	6.33E-03	33.01	32.52	0.50	32.30	30.79	1.51	1.01
cg04359840	XYLT1	xylosyltransferase I	206,167	346	6.52E-03	47.27	47.85	−0.58	47.80	49.99	−2.19	−1.61
cg21698310	PPP1R9B	protein phosphatase 1. regulatory subunit 15A	203,847	822	7.32E-03	10.66	10.89	−0.23	9.69	10.49	−0.81	−0.58
cg00816037	FAM38A	family with sequence similarity 38. member A	197,944	281	9.10E-03	7.77	8.09	−0.33	6.69	7.77	−1.08	−0.75
cg24488469			192,915	314	1.11E-02	15.97	16.30	−0.33	14.06	15.16	−1.09	−0.76
cg12033822	SLC35C2	solute carrier family 35 (GDP-fucose transporter). member C2	189,184	1.519	1.34E-02	26.76	27.15	−0.40	25.56	26.54	−0.98	−0.58
cg22490254			186,085	396	1.45E-02	15.92	15.44	0.48	15.51	13.87	1.64	1.16
cg26840970	ZNF19	zinc finger protein 19	315,231	309	1.54E-02	69.05	68.76	0.29	71.08	68.75	2.33	2.04
cg09608073	CHSY3	chondroitin sulfate synthase 3	184,657	712	1.55E-02	9.31	9.50	−0.19	7.99	8.47	−0.48	−0.29
cg19925780			179,222	134	1.85E-02	57.36	56.05	1.30	59.58	56.04	3.54	2.24
cg25223634	C10orf26	chromosome 10 open reading frame 26	172,484	368	1.90E-02	37.56	38.21	−0.64	35.14	37.58	−2.43	−1.79
cg00026474	ST3GAL1	ST3 beta-galactoside alpha-2.3-sialyltransferase 1	178,373	842	1.96E-02	19.10	19.54	−0.44	19.23	20.63	−1.40	−0.96
cg25114611	FKBP5	FK506 binding protein 5	362,777	198	1.97E-02	30.27	30.36	−0.08	30.44	31.81	−1.37	−1.28
cg01609214	MIR30D	microRNA 30d	174,169	418	2.24E-02	89.84	89.03	0.81	90.88	87.38	3.50	2.69
cg21188533	CACNA1D	calcium channel. voltage-dependent. L type. alpha 1D subunit	173,743	61	2.24E-02	43.74	41.82	1.91	52.19	43.74	8.45	6.54
cg06627354	TRPM8	transient receptor potential cation channel. subfamily M. member 8	173,046	285	2.32E-02	65.91	65.21	0.70	64.45	61.66	2.79	2.10
cg25722983	STK40	serine/threonine kinase 40	290,462	740	2.36E-02	45.26	45.55	−0.29	46.40	47.91	−1.52	−1.22
cg25233339	ATP1B3	ATPase. Na+/K+ transporting. beta 3 polypeptide	250,770	212	2.37E-02	3.69	3.79	−0.10	3.03	3.48	−0.45	−0.35
cg00449189			171,418	702	2.49E-02	12.06	12.39	−0.33	10.75	11.51	−0.77	−0.44
cg08287903	UGT8	UDP glycosyltransferase 8	169,875	180	2.58E-02	48.84	48.15	0.69	51.00	48.24	2.76	2.06
cg05055821			168,762	322	2.69E-02	9.90	9.65	0.26	9.44	8.67	0.77	0.52
cg05525812			171,414	3.675	2.76E-02	16.95	17.39	−0.44	15.71	16.55	−0.84	−0.40
cg20530056	IKBKE	inhibitor of kappa light polypeptide gene enhancer in B-cells. kinase epsilon	168,953	1.699	2.80E-02	63.75	64.26	−0.51	64.64	65.97	−1.33	−0.82
cg01963224			166,963	102	2.84E-02	12.83	13.10	−0.28	11.88	12.94	−1.06	−0.78
cg05548393	SLC30A8	solute carrier family 30 (zinc transporter). member 8	165,967	483	2.98E-02	77.09	76.22	0.87	78.67	76.30	2.37	1.50
cg24715767	PRDM2	PR domain containing 2. with ZNF domain	165,652	337	3.00E-02	57.80	57.13	0.67	61.48	59.19	2.29	1.62
cg05603910	ANO9	anoctamin 9	167,628	3.304	3.10E-02	78.58	77.55	1.03	75.77	73.98	1.79	0.77
cg16660971	RPTOR	regulatory associated protein of MTOR. complex 1	163,969	323	3.17E-02	72.09	71.01	1.08	71.01	66.95	4.06	2.98
cg06361984	NDE1	nudE neurodevelopment protein 1	159,841	809	3.69E-02	82.89	82.22	0.67	81.19	79.42	1.77	1.10
cg17873451	LOC440925		157,993	231	3.84E-02	4.11	4.26	−0.15	3.54	3.90	−0.37	−0.22
cg03220447	NAV2	neuron navigator 2	159,803	2.277	3.87E-02	12.43	12.70	−0.27	12.44	12.97	−0.53	−0.26
cg05756780	IL6R	interleukin 6 receptor	156,614	1.147	4.13E-02	23.32	23.63	−0.31	22.65	23.65	−1.00	−0.69
cg02104644	SYT7	synaptotagmin VII	158,677	4.255	4.27E-02	19.47	20.07	−0.59	18.63	19.87	−1.24	−0.64
cg08242636	CBFB	core-binding factor. beta subunit	155,174	1.149	4.33E-02	5.10	5.25	−0.15	5.00	5.37	−0.37	−0.22
cg22006825	HNRNPUL1	heterogeneous nuclear ribonucleoprotein U-like 1	171,009	17.624	4.41E-02	19.13	19.57	−0.45	19.20	19.75	−0.55	−0.10
cg07201017	FLJ41350	LBX1 antisense RNA 1 (head to head)	159,918	6.965	4.48E-02	16.26	16.59	−0.33	15.82	16.34	−0.52	−0.19
cg02571448	PCBP3	poly(rC) binding protein 3	151,595	974	4.81E-02	41.12	41.91	−0.79	41.66	43.59	−1.93	−1.14
cg24968629	CELSR1	cadherin. EGF LAG seven-pass G-type receptor 1	177,517	192	4.85E-02	74.90	73.75	1.15	70.52	65.45	5.07	3.92
cg25474070	IL3	interleukin 3	173,116	895	4.92E-02	62.86	63.34	−0.47	64.15	65.74	−1.59	−1.12
cg19859980	C1orf97	chromosome 1 open reading frame 97	154,094	4.225	4.92E-02	2.51	2.60	−0.09	2.59	2.74	−0.15	−0.06
cg03699074	FAM38A	family with sequence similarity 38. member A	150,341	568	4.94E-02	14.23	14.69	−0.46	12.81	14.07	−1.26	−0.79

^*^Methylation level (%); ^#^Δβ = difference in % methylation of current-never smokers.

**Table 4:** Female-specific DEGs/DMGs of potential relevance to CVD. and their expression/methylation changes observed in present study or reported clinical studies (where available).

Gene	Direct evidence of links with CVD pathogenesis	Mechanistic basis of links with CVD	Current study		Vasclular inflam-mation^28^	Chronic artery occlussion^48^	Myocardial infarction^63^	Coronary heart disease^64^	Coronary artery disease^65^	Peripheral arterial disease^66^	Myocardial infarction^67^	Venous thrombo-embolism^68^	Atherosus-ceptibility^69^
			expr*	meth#	expr*	expr*	expr*	expr*	expr*	expr*	expr*	expr*	meth#
F2R (PAR1)	polymorphisms in human CVD^14^	thrombin signaling/vascular haemostasis	down					down			down		
GP5				up									
FYN	transgenic mice^19^			down								up	
IGF1R				up									up
EGF				up									
EPS8	transgenic mice^24^			up									
RPTOR	transgenic mice^26^			up									
HMOX1	polymorphisms and differential expression in human CVD^28^; transgenic mice^28^	anti-inflammatory	down		down								
HDAC4		regulation of hypertrophy											
BCL2L1		anti-apoptotic		down				up					
CACNA1D	polymorphisms in human CVD^37^; transgenic mice^35^,^36^	calcium signalling		up									
TANGLN	polymorphisms in human CVD^70^; transgenic mice^42^	VSMC/endothelial cell function	down										
SYNE1	polymorphisms in human CVD^37^; transgenic mice^35^,^36^		down								down		
IL32	differential expression in human CVD^45^; transgenic mice^45^	pro-inflammatory		down									
PLIN5	transgenic mice^46^	oxidative stress		down									
HNRNPUL1	polymorphisms in human CVD^47^	unknown		down									
miR-30d	differential expression in human CVD^48^	regulation of cardiomyocyte apoptosis^71^		up		up	down						
C14orf43	differential expression in human CVD^61^	unknown		down						down			
C1orf21	differential expression in human CVD^59^	unknown	down					down					
ST3GAL1	differential expression in human CVD^59^	unknown		down				up					
ZNF19	differential expression in human CVD^59^	unknown		up				down					

The genes are listed in the order in which they are discussed in the main text. Col. 1 presents evidence based on human population and transgenic animal studies.

^*^Expression; ^#^methylation.
