# Supplementary material for: Blood-based omic profiling supports female susceptibility to tobacco smoke-induced cardiovascular diseases
Source: Sci Rep. 2017 Feb 22;7:42870. doi: 10.1038/srep42870 (PMC5320491; doi:10.1038/srep42870)

SUPPLEMENTARY INFORMATION

Blood-based omic profiling supports female susceptibility to tobacco smoke-induced cardiovascular diseases

Authors: Aristotelis Chatziioannou, Panagiotis Georgiadis, Dennie G. Hebels, Irene Liampa, Ioannis Valavanis, Ingvar A. Bergdahl, Anders Johansson, Domenico Palli, Marc Chadeau-Hyam, Alexandros P. Siskos, Hector Keun, Maria Botsivali, Theo M.C.M. de Kok, Almudena Espín Pérez, Jos C.S. Kleinjans, Paolo Vineis and Soterios A. Kyrtopoulos, the EnviroGenomarkers project consortium

Supplementary Table S1: Probes and associated genes differentially expressed in current smokers (FDR<0.10)

probe no.	gene	raw p value	FDR	foldchange expression
A_24_P187766	LRRN3	<1E-17	<1E-17	1.95
A_23_P64611	P2RY6	<1E-17	<1E-17	1.38
A_23_P31376	LRRN3	<1E-17	<1E-17	1.96
A_23_P428129	CDKN1C	4.85E-14	3.60E-10	0.65
A_23_P115785	FANK1	1.39E-11	8.23E-08	1.43
A_24_P904484	LOC283174	4.74E-10	2.34E-06	1.32
A_23_P150789	PRSS23	2.13E-09	9.03E-06	0.70
A_24_P937405	PRSS23	8.17E-09	3.03E-05	0.70
A_32_P63013	LOC283174	1.16E-08	3.81E-05	1.32
A_23_P61042		3.26E-08	9.09E-05	1.57
A_23_P138492	NEURL	3.37E-08	9.09E-05	0.82
A_23_P26386	TPPP3	4.46E-08	1.00E-04	0.75
A_23_P211680	MLC1	4.80E-08	1.00E-04	0.77
A_23_P87421		6.09E-08	1.00E-04	0.71
A_23_P253375	CUX1	7.75E-08	2.00E-04	0.84
A_24_P511686	LOC100506870	1.03E-07	2.00E-04	1.23
A_23_P12514	RHOC	1.19E-07	2.00E-04	0.83
A_23_P53081	OSBPL5	1.37E-07	2.00E-04	0.79
A_23_P93442	SASH1	1.41E-07	2.00E-04	1.32
A_23_P371276	C18orf23	1.69E-07	3.00E-04	0.76
A_23_P358709	AHRR	1.96E-07	3.00E-04	1.73
A_23_P40847	CHST2	2.55E-07	3.00E-04	0.80
A_23_P325562	SLC1A7	2.94E-07	4.00E-04	0.76
A_23_P160720	BATF3	3.23E-07	4.00E-04	0.81
A_23_P351148	SH2D1B	3.68E-07	4.00E-04	0.75
A_24_P300379	PI16	3.99E-07	5.00E-04	1.22
A_24_P245838	MGAT3	4.18E-07	5.00E-04	1.34
A_23_P119042	NKG7	4.82E-07	5.00E-04	0.81
A_23_P379736	B3GNT9	4.84E-07	5.00E-04	1.15
A_23_P74088	MMP23B	5.85E-07	6.00E-04	0.82
A_24_P265523	CR1	6.79E-07	6.00E-04	1.26
A_23_P107744	S1PR5	8.32E-07	8.00E-04	0.75
A_32_P930685	ZNF876P	8.38E-07	8.00E-04	0.80
A_23_P433760	SPN	1.02E-06	9.00E-04	0.88
A_24_P465799		1.37E-06	1.20E-03	1.43
A_23_P373100	MGC24103	1.50E-06	1.20E-03	0.66
A_23_P254193	TTC38	1.71E-06	1.40E-03	0.80
A_23_P209700	NMUR1	1.82E-06	1.40E-03	0.79
A_23_P254654	CLIC3	2.00E-06	1.50E-03	0.80
A_32_P324533	SH2D1B	2.30E-06	1.70E-03	0.76
A_23_P152559	BZRAP1	2.31E-06	1.70E-03	0.83
A_23_P206284	GPR56	2.34E-06	1.70E-03	0.81
A_32_P171061	ASCL2	2.84E-06	1.90E-03	0.82
A_23_P3083		2.89E-06	1.90E-03	0.75
A_23_P206280	GPR56	3.01E-06	2.00E-03	0.76
A_32_P190461		3.07E-06	2.00E-03	1.26
A_23_P43157	MYBL1	3.12E-06	2.00E-03	0.81
A_32_P158966	KLRF1	3.18E-06	2.00E-03	0.78
A_23_P41528	FGFBP2	3.57E-06	2.20E-03	0.74
A_24_P226069	FGFBP2	3.89E-06	2.30E-03	0.75
A_23_P146554	PTGDS	4.26E-06	2.50E-03	0.81
A_24_P921823	TCF7L2	4.80E-06	2.70E-03	0.83
A_23_P102113	WNT10A	5.24E-06	2.90E-03	1.16
A_23_P94186	LYPD2	5.44E-06	3.00E-03	0.79
A_23_P133445	GZMA	5.95E-06	3.20E-03	0.80
A_32_P133916	BNC2	6.06E-06	3.20E-03	0.73
A_24_P108311	NEDD4L	6.08E-06	3.20E-03	1.18
A_24_P169873		6.27E-06	3.20E-03	1.46

A_23_P108042	NCR1	6.99E-06	3.50E-03	0.79
A_23_P108404	AGAP1	7.27E-06	3.50E-03	0.81
A_23_P136026		7.31E-06	3.50E-03	1.33
A_23_P122662	GFOD1	7.31E-06	3.50E-03	0.84
A_24_P79403	PF4	8.82E-06	4.20E-03	0.76
A_32_P429876	PPM1L	9.57E-06	4.40E-03	0.80
A_24_P88850	MRAS	1.04E-05	4.70E-03	0.81
A_23_P203351	MS4A7	1.05E-05	4.70E-03	0.83
A_23_P128230	NR4A1	1.07E-05	4.70E-03	0.63
A_32_P15035	GFOD1	1.22E-05	5.30E-03	0.84
A_23_P141555	TBX21	1.25E-05	5.40E-03	0.80
A_23_P372496	DNAH12	1.29E-05	5.50E-03	0.71
A_23_P155556	CLDND1	1.35E-05	5.60E-03	1.15
A_32_P164593	ZMAT4	1.50E-05	6.10E-03	0.70
A_23_P50946	RAMP1	1.51E-05	6.10E-03	0.81
A_23_P16976	ANXA4	1.56E-05	6.30E-03	0.87
A_23_P121596	PPBP	1.71E-05	6.70E-03	0.74
A_23_P61987	TMEM121	1.72E-05	6.70E-03	1.17
A_23_P120883	HMOX1	1.77E-05	6.80E-03	0.84
A_23_P70719	LAMA2	1.85E-05	7.00E-03	0.82
A_23_P254507	HOPX	1.96E-05	7.20E-03	0.85
A_24_P154080	ECE1	1.97E-05	7.20E-03	1.18
A_24_P519504		1.97E-05	7.20E-03	1.36
A_24_P353619	ALPL	2.11E-05	7.50E-03	1.26
A_23_P121533	SPON2	2.11E-05	7.50E-03	0.77
A_23_P110941	GSTA4	2.18E-05	7.60E-03	0.87
A_23_P406424	RHOC	2.19E-05	7.60E-03	0.91
A_24_P156388	TTC38	2.60E-05	9.00E-03	0.82
A_24_P913146	HOPX	2.65E-05	9.10E-03	0.84
A_23_P136753		3.09E-05	1.00E-02	1.20
A_23_P381714	CA13	3.10E-05	1.00E-02	0.84
A_23_P346900	CACNA2D2	3.12E-05	1.00E-02	0.84
A_23_P26994	GNGT2	3.13E-05	1.00E-02	0.86
A_23_P354341	CD160	3.14E-05	1.00E-02	0.78
A_32_P87191	FLJ21408	3.14E-05	1.00E-02	1.29
A_23_P56578	VIT	3.23E-05	1.02E-02	0.76
A_23_P117602	GZMB	3.26E-05	1.02E-02	0.80
A_23_P21485	PID1	3.36E-05	1.04E-02	1.18
A_23_P138117	CAMTA1	3.42E-05	1.05E-02	0.87
A_23_P97046	ALPL	3.45E-05	1.05E-02	1.22
A_32_P133564		3.61E-05	1.08E-02	1.25
A_23_P1833	B3GAT1	3.73E-05	1.10E-02	0.77
A_24_P175435	SLC2A8	3.76E-05	1.10E-02	0.88
A_23_P397671	CR1	3.97E-05	1.15E-02	1.23
A_32_P12232	BNC2	4.04E-05	1.16E-02	0.72
A_24_P173754	C1orf21	4.36E-05	1.24E-02	0.82
A_24_P240166	PHLDB2	4.50E-05	1.27E-02	0.84
A_24_P395415		4.67E-05	1.30E-02	1.30
A_23_P253221	ARHGEF4	4.69E-05	1.30E-02	1.16
A_23_P4494	DSC2	4.78E-05	1.31E-02	1.34
A_24_P362805	GK5	4.93E-05	1.34E-02	0.86
A_23_P406385	FBXL16	4.99E-05	1.35E-02	1.17
A_32_P69368	ID2	5.27E-05	1.41E-02	0.86
A_32_P26721		5.59E-05	1.48E-02	0.88
A_32_P166693	HEG1	5.74E-05	1.51E-02	0.87
A_23_P112482	AQP3	5.96E-05	1.55E-02	1.16
A_23_P1331	COL13A1	6.03E-05	1.55E-02	0.76
A_23_P113748	ZNF385D	6.05E-05	1.55E-02	0.76
A_23_P57347	PCNT	6.22E-05	1.58E-02	0.90
A_23_P213102	PALLD	6.55E-05	1.65E-02	0.83
A_23_P200780	TGFBR3	7.03E-05	1.75E-02	0.83
A_24_P272993	MEG3	7.11E-05	1.75E-02	0.73
A_24_P203000	IL2RB	7.16E-05	1.75E-02	0.84

A_23_P202881	FEZ1	7.24E-05	1.75E-02	0.82
A_24_P658584	SASH1	7.28E-05	1.75E-02	1.31
A_24_P230563	IL2RA	7.30E-05	1.75E-02	1.18
A_23_P418015	MAPRE2	7.50E-05	1.77E-02	0.88
A_23_P69171	SUCNR1	7.50E-05	1.77E-02	0.77
A_24_P417706	MXD3	7.81E-05	1.81E-02	1.20
A_23_P23834	LGR6	7.87E-05	1.81E-02	0.78
A_23_P136347	EPS8	7.87E-05	1.81E-02	0.81
A_23_P93524	SAMD3	8.11E-05	1.85E-02	0.83
A_23_P25674	CKB	8.37E-05	1.89E-02	0.84
A_23_P327380	TP63	8.41E-05	1.89E-02	1.27
A_23_P211039	ADAMTS1	8.59E-05	1.92E-02	0.79
A_24_P417460	SFMBT2	8.75E-05	1.94E-02	0.81
A_23_P113161	C1orf21	9.05E-05	1.99E-02	0.80
A_32_P129310		9.21E-05	2.01E-02	1.15
A_23_P32233	KLF4	9.41E-05	2.04E-02	0.85
A_23_P33768	ZFYVE9	9.98E-05	2.13E-02	1.16
A_24_P13041	RTKN2	9.99E-05	2.13E-02	1.24
A_23_P143143	ID2	1.00E-04	2.13E-02	0.85
A_24_P113131	BZRAP1	1.00E-04	2.17E-02	0.84
A_32_P34404		1.00E-04	2.20E-02	0.86
A_23_P203920	SSPN	1.00E-04	2.20E-02	1.18
A_23_P366366	SCRN1	1.00E-04	2.29E-02	0.87
A_23_P146943	ATP1B1	1.00E-04	2.30E-02	0.84
A_23_P353436		1.00E-04	2.32E-02	0.87
A_24_P583225	NCRNA00265	1.00E-04	2.42E-02	1.18
A_23_P44505	KLF11	1.00E-04	2.48E-02	0.87
A_23_P53193	SYTL2	1.00E-04	2.49E-02	0.83
A_24_P343233	HLA-DRB1	1.00E-04	2.49E-02	0.89
A_23_P138706	ADRA2A	1.00E-04	2.69E-02	0.81
A_23_P107750	S1PR5	1.00E-04	2.77E-02	0.80
A_23_P151120	ACRBP	1.00E-04	2.77E-02	0.81
A_23_P127948	ADM	1.00E-04	2.78E-02	1.30
A_23_P22096	PTK2	1.00E-04	2.81E-02	0.84
A_24_P399220	HOXB3	1.00E-04	2.82E-02	0.82
A_23_P16006	ZNF600	1.00E-04	2.82E-02	0.87
A_23_P257365	GFI1	2.00E-04	2.82E-02	0.82
A_24_P173566		2.00E-04	2.82E-02	1.24
A_24_P179816	SLC27A3	2.00E-04	2.83E-02	0.90
A_23_P72989	CCR4	2.00E-04	2.83E-02	1.18
A_23_P112452	GGTA1	2.00E-04	2.95E-02	0.78
A_32_P50406	FLJ45340	2.00E-04	3.03E-02	1.17
A_24_P184803	COCH	2.00E-04	3.03E-02	1.30
A_23_P98565	MS4A14	2.00E-04	3.11E-02	0.80
A_24_P24890		2.00E-04	3.27E-02	1.31
A_23_P72252		2.00E-04	3.29E-02	1.23
A_23_P200685	MOSC2	2.00E-04	3.31E-02	1.17
A_24_P234554	ZNF385D	2.00E-04	3.34E-02	0.73
A_23_P43684	BNC2	2.00E-04	3.43E-02	0.82
A_32_P196669		2.00E-04	3.43E-02	1.19
A_23_P51231	RUNX3	2.00E-04	3.45E-02	0.87
A_23_P8913	CA2	2.00E-04	3.47E-02	0.81
A_23_P86682	MYOF	2.00E-04	3.47E-02	0.83
A_24_P589028		2.00E-04	3.55E-02	0.73
A_23_P121676	CXXC4	2.00E-04	3.55E-02	0.76
A_23_P206290	GPR114	2.00E-04	3.55E-02	0.86
A_23_P43979		2.00E-04	3.55E-02	1.23
A_23_P66881	RGS9	2.00E-04	3.62E-02	0.81
A_23_P39251	PLIN5	2.00E-04	3.62E-02	1.23
A_23_P99275	KLRB1	2.00E-04	3.67E-02	0.85
A_23_P109508	NCF4	2.00E-04	3.72E-02	1.21
A_23_P385105	PLCD4	2.00E-04	3.88E-02	0.85
A_24_P267814	LOC100287891	2.00E-04	3.88E-02	1.26

A_23_P201376	SSX2IP	2.00E-04	3.90E-02	0.81
A_24_P743802	ZNF618	2.00E-04	3.90E-02	0.84
A_23_P39931	DYSF	2.00E-04	3.90E-02	1.17
A_24_P141481	CD59	3.00E-04	3.96E-02	1.10
A_23_P109171	BFSP1	3.00E-04	3.97E-02	0.82
A_23_P336612	C15orf26	3.00E-04	3.98E-02	0.78
A_23_P347632	MTSS1	3.00E-04	3.98E-02	0.88
A_23_P259506	C5orf32	3.00E-04	3.98E-02	1.18
A_23_P213620	PPP2R2B	3.00E-04	4.02E-02	0.86
A_23_P348257	NUAK1	3.00E-04	4.03E-02	0.80
A_23_P127128	DNAJC1	3.00E-04	4.03E-02	0.90
A_23_P99614	BTBD6	3.00E-04	4.03E-02	0.92
A_23_P48212	CLEC1B	3.00E-04	4.07E-02	0.79
A_24_P139604	PYHIN1	3.00E-04	4.07E-02	0.83
A_32_P75141		3.00E-04	4.07E-02	0.85
A_24_P339560	SIGLEC11	3.00E-04	4.07E-02	1.26
A_23_P328740	NEURL3	3.00E-04	4.09E-02	0.78
A_23_P89249	ERBB2	3.00E-04	4.09E-02	0.89
A_23_P374902	CLDND2	3.00E-04	4.19E-02	0.86
A_24_P383523	SAMD4A	3.00E-04	4.20E-02	0.89
A_23_P209527	VIL1	3.00E-04	4.28E-02	0.77
A_24_P466590		3.00E-04	4.28E-02	0.88
A_24_P104980	IGLL5	3.00E-04	4.28E-02	1.17
A_24_P119141	PROS1	3.00E-04	4.34E-02	0.75
A_23_P397937	SAMD3	3.00E-04	4.41E-02	0.85
A_24_P166443	HLA-DPB1	3.00E-04	4.50E-02	0.88
A_23_P13232		3.00E-04	4.53E-02	0.80
A_23_P157569	ADHFE1	3.00E-04	4.53E-02	0.87
A_23_P85453	CD244	3.00E-04	4.53E-02	0.88
A_24_P274219	EPHA4	3.00E-04	4.53E-02	1.14
A_24_P940166	PAPSS2	3.00E-04	4.55E-02	0.83
A_23_P397208	GSTM2	3.00E-04	4.55E-02	0.85
A_32_P25639	BET3L	3.00E-04	4.57E-02	0.79
A_23_P342275	ADAMTS1	3.00E-04	4.64E-02	0.80
A_23_P205200	DHRS12	3.00E-04	4.64E-02	1.14
A_24_P396167	CTSW	3.00E-04	4.67E-02	0.84
A_23_P216094	ASPH	3.00E-04	4.68E-02	1.14
A_23_P17633	IFNAR1	4.00E-04	4.70E-02	1.12
A_23_P434118	CEACAM1	4.00E-04	4.74E-02	1.16
A_23_P154526	GRB14	4.00E-04	4.78E-02	0.79
A_23_P214244	ENPP5	4.00E-04	4.78E-02	0.80
A_23_P163697	SYT17	4.00E-04	4.78E-02	0.86
A_23_P101093	COPZ2	4.00E-04	4.78E-02	0.86
A_23_P72050	PTK2	4.00E-04	4.78E-02	0.86
A_32_P163147	VSIG1	4.00E-04	4.78E-02	1.10
A_32_P150012	CHD7	4.00E-04	4.97E-02	1.12
A_24_P184799	COCH	4.00E-04	4.98E-02	1.32
A_23_P205370	ASB2	4.00E-04	5.04E-02	1.13
A_23_P31006	HLA-DRB5	4.00E-04	5.11E-02	0.86
A_24_P414169	TFDP2	4.00E-04	5.17E-02	0.89
A_24_P129632	DLG5	4.00E-04	5.22E-02	0.80
A_23_P128728	ARG2	4.00E-04	5.22E-02	0.81
A_23_P19987	IGF2BP3	4.00E-04	5.22E-02	0.82
A_23_P104493	PAPSS2	4.00E-04	5.22E-02	0.84
A_23_P400378	GPBAR1	4.00E-04	5.22E-02	0.89
A_23_P115417	RGL1	4.00E-04	5.22E-02	1.11
A_32_P139163		4.00E-04	5.22E-02	1.15
A_23_P156826	C6orf105	4.00E-04	5.22E-02	1.21
A_23_P256821	CR1	4.00E-04	5.22E-02	1.22
A_23_P421323		4.00E-04	5.22E-02	1.28
A_23_P24193	XPNPEP1	4.00E-04	5.25E-02	0.88
A_23_P138541	AKR1C3	4.00E-04	5.33E-02	0.81
A_23_P158925	GPR125	5.00E-04	5.60E-02	0.80

A_23_P380614	ATP9A	5.00E-04	5.60E-02	0.83
A_23_P75325	SGPL1	5.00E-04	5.60E-02	0.88
A_23_P435390		5.00E-04	5.60E-02	1.27
A_23_P412214	RAP1GAP2	5.00E-04	5.61E-02	0.78
A_23_P56213	GRAMD1A	5.00E-04	5.61E-02	1.11
A_24_P342178		5.00E-04	5.66E-02	1.12
A_23_P48175	TMEM106C	5.00E-04	5.72E-02	0.88
A_23_P1782	CD82	5.00E-04	5.72E-02	1.11
A_23_P413456	CIRBP	5.00E-04	5.72E-02	1.18
A_23_P123413	TOX	5.00E-04	5.73E-02	0.84
A_32_P88120	YPEL1	5.00E-04	5.75E-02	0.90
A_24_P472081		5.00E-04	5.75E-02	1.18
A_32_P156786		5.00E-04	5.75E-02	1.22
A_23_P155939	ZNF595	5.00E-04	6.03E-02	0.84
A_32_P89269		5.00E-04	6.05E-02	0.81
A_23_P418373	BCL2L2	5.00E-04	6.09E-02	0.89
A_23_P170534	FUT7	5.00E-04	6.12E-02	1.15
A_23_P397391	FFAR2	5.00E-04	6.12E-02	1.22
A_23_P167168	IGJ	5.00E-04	6.12E-02	1.24
A_24_P83102	IGLL1	6.00E-04	6.13E-02	1.26
A_24_P565503		6.00E-04	6.19E-02	1.14
A_32_P43664		6.00E-04	6.19E-02	1.29
A_24_P409971	NEXN	6.00E-04	6.26E-02	0.82
A_24_P88763	LOXL3	6.00E-04	6.28E-02	0.89
A_23_P52451	HKDC1	6.00E-04	6.49E-02	1.17
A_24_P940288	PGS1	6.00E-04	6.49E-02	1.18
A_23_P1473	PRF1	6.00E-04	6.52E-02	0.82
A_24_P410453	SYNE1	6.00E-04	6.52E-02	0.84
A_24_P246573	KAZ	6.00E-04	6.68E-02	1.31
A_23_P34045	EDA	6.00E-04	6.92E-02	1.13
A_23_P58796	RGMB	6.00E-04	6.92E-02	1.23
A_23_P168828	KLF10	7.00E-04	6.92E-02	0.84
A_32_P140139	F13A1	7.00E-04	6.96E-02	0.82
A_24_P178602	ZNF600	7.00E-04	6.96E-02	0.85
A_23_P2661	RAP1B	7.00E-04	6.96E-02	0.90
A_24_P298877	C1orf174	7.00E-04	6.96E-02	0.91
A_24_P302406		7.00E-04	6.97E-02	0.90
A_24_P76644		7.00E-04	7.11E-02	0.90
A_23_P38830	ZNF552	7.00E-04	7.12E-02	1.14
A_23_P166306	CBS	7.00E-04	7.17E-02	1.33
A_23_P43107	TM7SF4	7.00E-04	7.18E-02	0.80
A_23_P17095	TFPI	7.00E-04	7.54E-02	0.82
A_23_P132226	TPST2	7.00E-04	7.60E-02	0.90
A_23_P218225	QPRT	7.00E-04	7.61E-02	0.92
A_23_P119794		8.00E-04	7.65E-02	0.86
A_23_P211550	RBX1	8.00E-04	7.65E-02	0.92
A_23_P386241	FAM110A	8.00E-04	7.66E-02	0.91
A_23_P88351	ATL1	8.00E-04	7.80E-02	0.88
A_23_P159237	GPR20	8.00E-04	7.86E-02	0.87
A_24_P755069		8.00E-04	7.90E-02	0.85
A_23_P13031	CTSW	8.00E-04	8.06E-02	0.86
A_23_P45831	CHD1L	8.00E-04	8.06E-02	0.92
A_23_P112957		8.00E-04	8.06E-02	1.13
A_23_P100704	MAPK7	8.00E-04	8.08E-02	0.92
A_23_P302550	RGS18	8.00E-04	8.09E-02	0.82
A_23_P43679	ZNF618	8.00E-04	8.09E-02	0.89
A_23_P3450	TUBGCP4	8.00E-04	8.09E-02	0.92
A_23_P312932	KRTAP8-1	8.00E-04	8.09E-02	1.20
A_24_P272222	PLIN5	8.00E-04	8.12E-02	1.16
A_23_P209129	LAIR2	8.00E-04	8.17E-02	0.73
A_23_P139486	CDK2AP1	8.00E-04	8.17E-02	0.89
A_23_P9485	ORM2	9.00E-04	8.30E-02	1.22
A_23_P57413	PPM1F	9.00E-04	8.43E-02	1.13

A_23_P34915	ATF3	9.00E-04	8.47E-02	0.78
A_23_P428887	KLHL34	9.00E-04	8.47E-02	1.12
A_23_P116512	PRR5L	9.00E-04	8.55E-02	0.83
A_32_P140475	KIAA1377	9.00E-04	8.66E-02	0.88
A_23_P212779	PARM1	9.00E-04	8.66E-02	1.14
A_32_P172848	GK	9.00E-04	8.66E-02	1.15
A_32_P90615		9.00E-04	8.66E-02	1.18
A_32_P54289		9.00E-04	8.72E-02	1.19
A_23_P1962	RARRES3	9.00E-04	8.74E-02	0.90
A_32_P187009	SERINC5	9.00E-04	8.75E-02	1.12
A_24_P367227	MYBL1	1.00E-03	9.03E-02	0.84
A_23_P377267		1.00E-03	9.03E-02	0.90
A_23_P66719	DHRS13	1.00E-03	9.03E-02	1.14
A_23_P40174	MMP9	1.00E-03	9.03E-02	1.23
A_32_P32195		1.00E-03	9.05E-02	0.87
A_23_P217510	CD99	1.00E-03	9.05E-02	0.91
A_23_P148768	F5	1.00E-03	9.05E-02	1.15
A_24_P355693	ACER3	1.00E-03	9.23E-02	0.86
A_23_P101407	C3	1.00E-03	9.24E-02	0.89
A_23_P40548	YPEL1	1.00E-03	9.24E-02	0.91
A_32_P220897		1.00E-03	9.40E-02	1.11
A_24_P124349	PDGFD	1.10E-03	9.56E-02	0.80
A_32_P28158		1.10E-03	9.70E-02	1.18
A_23_P87013	TAGLN	1.10E-03	9.71E-02	0.86
A_32_P141768	AGPAT4	1.10E-03	9.71E-02	0.88
A_23_P135123		1.10E-03	9.77E-02	0.82
A_24_P353638	SLAMF7	1.10E-03	9.77E-02	0.87
A_24_P266734	SSH3	1.10E-03	9.77E-02	1.09
A_23_P209625	CYP1B1	1.10E-03	9.77E-02	1.19
A_23_P211910	PLOD2	1.10E-03	9.79E-02	0.80
A_24_P9883	DKFZp761E198	1.10E-03	9.79E-02	1.22
A_23_P354387	MYOF	1.10E-03	9.80E-02	0.85
A_23_P39925	DYSF	1.10E-03	9.80E-02	1.17
A_23_P45917	CKS1B	1.10E-03	9.85E-02	0.91
A_24_P215240	ENKUR	1.20E-03	9.85E-02	0.80
A_23_P110791	CSF1R	1.20E-03	9.85E-02	0.87
A_24_P167642	GCH1	1.20E-03	9.85E-02	0.88
A_23_P130836	GZMM	1.20E-03	9.85E-02	0.91
A_24_P169843		1.20E-03	9.85E-02	1.16
A_23_P43810	LTBP1	1.20E-03	9.99E-02	0.79

Supplementary Table S2: CpG sites and associated genes differentially methylated in current smokers (FDR-corrected p<0.05)

CpG site	gene	raw p value	FDR	β (methylation). smokers (%)	β (methylation). never smokers (%)	Δβ
cg05575921	AHRR	8.1E-87	3.33E-81	53.72	78.06	-24.34
cg21566642		1.52E-70	3.12E-65	29.85	44.88	-15.03
cg03636183	F2RL3	2.8E-67	3.83E-62	55.04	65.85	-10.81
cg01940273		1.38E-64	1.42E-59	47.05	57.5	-10.45
cg05951221		7.13E-60	5.86E-55	28.78	39.28	-10.51
cg06126421		7.27E-43	4.27E-38	52.23	62.46	-10.23
cg26703534	AHRR	1.64E-37	8.41E-33	57.34	62.53	-5.19
cg25648203	AHRR	9.41E-33	4.3E-28	71.73	76.3	-4.57
cg15342087		3.41E-32	1.4E-27	76.07	79.98	-3.91
cg03329539		2.74E-31	1.02E-26	29.03	33.44	-4.40
cg19859270	GPR15	1.78E-28	6.1E-24	70.35	74.91	-4.56
cg09935388	GFI1	8.36E-26	2.64E-21	61.19	69.18	-7.98
cg14753356		1E-25	2.94E-21	29.36	33.25	-3.90
cg27537125		2.17E-25	5.94E-21	10.32	11.9	-1.58
cg14817490	AHRR	4.7E-25	1.21E-20	16.83	20.93	-4.10
cg24859433		2.75E-23	6.65E-19	74.97	78.58	-3.61
cg21611682	LRP5	5.49E-23	1.25E-18	48.85	51.86	-3.01
cg22132788	MYO1G	9.41E-23	2.03E-18	87.8	73.93	13.87
cg19572487	RARA	1.99E-22	4.1E-18	43.24	48.25	-5.01
cg06644428		2.19E-22	4.28E-18	2.74	4.31	-1.57
cg23079012		3E-22	5.61E-18	85.99	94.63	-8.64
cg12803068	MYO1G	9.56E-22	1.71E-17	74.56	62.41	12.14
cg23161492	ANPEP	1.15E-21	1.97E-17	18.05	22.5	-4.45
cg25949550	CNTNAP2	2.78E-21	4.58E-17	7.12	8.61	-1.49
cg23576855	AHRR	4.28E-21	6.77E-17	41.15	57.24	-16.09
cg21322436	CNTNAP2	2.23E-20	3.39E-16	19	21.84	-2.84
cg11660018	PRSS23	5.08E-20	7.46E-16	43.83	47.97	-4.14
cg04885881		8.45E-20	1.2E-15	31.14	35.38	-4.23
cg11902777	AHRR	2.03E-19	2.78E-15	2.77	3.78	-1.00
cg27241845		3.98E-18	5.28E-14	52.99	57.14	-4.15
cg24996979	C14orf43	1.61E-17	2.06E-13	16.8	18.6	-1.79
cg03707168	PPP1R15A	2.03E-17	2.53E-13	18.9	21.8	-2.90
cg23916896	AHRR	2.11E-17	2.56E-13	13.82	17.69	-3.87
cg11207515	CNTNAP2	5.26E-17	6.18E-13	34.98	30.15	4.83
cg25189904	GNG12	1.23E-16	1.4E-12	36.65	43.32	-6.67
cg04551776	AHRR	1E-15	1.11E-11	69.69	72.48	-2.79
cg01692968		2.89E-15	3.13E-11	21.86	24.7	-2.84
cg07339236	ATP9A	3.11E-15	3.27E-11	7.46	9.06	-1.60
cg23771366	PRSS23	4.27E-15	4.39E-11	38.23	41.6	-3.37
cg03450842	ZMIZ1	9.29E-15	9.31E-11	54.63	57.26	-2.62
cg16145216	HIVEP3	1.34E-14	1.31E-10	29.74	25.74	3.99
cg17287155	AHRR	1.91E-14	1.83E-10	81.7	84.66	-2.95

cg12876356	GFI1	2.09E-14	1.93E-10	65.52	70.94	-5.42
cg00310412	SEMA7A	2.11E-14	1.93E-10	44.26	47.18	-2.93
cg20295214	AVPR1B	4E-14	3.57E-10	60.49	63.71	-3.21
cg03991871	AHRR	5.86E-14	5.12E-10	68	73.54	-5.54
cg04180046	MYO1G	8.49E-14	7.27E-10	40.98	36.4	4.58
cg12513616		8.74E-14	7.33E-10	38.75	41.32	-2.57
cg02657160	CPOX	1.14E-13	9.4E-10	78.1	80.16	-2.06
cg00073090		1.51E-13	1.21E-09	33.42	35.8	-2.37
cg12806681	AHRR	2E-13	1.58E-09	71.51	75.24	-3.74
cg15542713	HIVEP3	3.16E-13	2.45E-09	44.32	37.95	6.37
cg24049493	HIVEP3	1.76E-12	1.34E-08	19.33	14.97	4.36
cg01257799	CXCR5	1.79E-12	1.34E-08	12.74	13.77	-1.03
cg19089201	MYO1G	2.5E-12	1.84E-08	80.87	73.34	7.54
cg16611234		2.66E-12	1.92E-08	23.68	26.75	-3.07
cg02532700	NCF4	4E-12	2.83E-08	13.75	16.22	-2.48
cg02451831	KIAA0087	4.14E-12	2.88E-08	67.21	69.4	-2.19
cg14624207	LRP5	4.44E-12	3.04E-08	47.33	49.65	-2.32
cg01731783	C14orf43	5.06E-12	3.41E-08	56.42	58.7	-2.28
cg26361535	ZC3H3	7.81E-12	5.18E-08	63.94	67.65	-3.71
cg17619755	VARS	9.92E-12	6.47E-08	58.68	54.42	4.26
cg06635952	ANXA4	1.26E-11	8.06E-08	25.72	23.94	1.78
cg21733098		1.32E-11	8.36E-08	56.21	62.27	-6.06
cg01901332	ARRB1	1.35E-11	8.41E-08	60.04	62.99	-2.95
cg18754985	CLDND1	1.38E-11	8.47E-08	85.16	87.16	-2.00
cg11554391	AHRR	1.62E-11	9.8E-08	11.85	13.54	-1.69
cg23973524	CRTC1	2.16E-11	1.29E-07	53.44	49.59	3.85
cg15159987	CPAMD8	2.71E-11	1.58E-07	55.17	57.55	-2.38
cg23480021		2.73E-11	1.58E-07	46.99	37.05	9.93
cg18146737	GFI1	3.23E-11	1.84E-07	55.74	71.1	-15.36
cg12075928	PTK2	3.38E-11	1.9E-07	39.98	43.14	-3.16
cg09099830	ITGAL	3.67E-11	2.04E-07	47.3	50.32	-3.01
cg20059012	RARG	4.52E-11	2.48E-07	2.49	3.72	-1.23
cg06235438	ITGAL	4.9E-11	2.65E-07	65.81	68.21	-2.40
cg10750182	C10orf105	5.11E-11	2.69E-07	50.41	52.27	-1.85
cg18316974	GFI1	5.15E-11	2.69E-07	72.33	81.38	-9.05
cg13751113	AMICA1	5.16E-11	2.69E-07	14.29	15.61	-1.32
cg13039251	PDZD2	5.23E-11	2.69E-07	68.79	64.09	4.70
cg07826859	MYO1G	6.48E-11	3.29E-07	50.64	52.89	-2.25
cg24090911	AHRR	1.48E-10	7.42E-07	57.96	61.43	-3.47
cg21473814	CRTC1	1.99E-10	9.86E-07	63.46	60.3	3.16
cg12593793		2.16E-10	1.06E-06	22.81	24.49	-1.68
cg11094248	RARA	2.25E-10	1.08E-06	10.64	11.57	-0.93
cg07202214	LRRC32	2.26E-10	1.08E-06	22.68	24.58	-1.90
cg26271591	NFE2L2	2.74E-10	1.28E-06	27.5	30.92	-3.43
cg08709672	AVPR1B	2.75E-10	1.28E-06	53.33	55.98	-2.65
cg25292882		2.81E-10	1.3E-06	73.23	75.73	-2.50
cg25909396	PRKCA	4.4E-10	2.01E-06	73.6	75.84	-2.25
cg20533899	LRRC32	4.72E-10	2.13E-06	17.9	19.38	-1.48
cg03274391		5.1E-10	2.28E-06	46.53	38.9	7.63

cg19827923	GPR55	5.77E-10	2.55E-06	73.71	75.44	-1.73
cg24908166	TERT	6.35E-10	2.78E-06	82.6	85.71	-3.12
cg10874644		6.43E-10	2.78E-06	58.09	53.9	4.19
cg01554474	RAG1AP1	6.8E-10	2.91E-06	20.42	22.49	-2.07
cg21393163		7.1E-10	3.01E-06	7.72	9.14	-1.43
cg13193840		7.31E-10	3.06E-06	3.93	4.64	-0.71
ch.1.171672612F		8.58E-10	3.56E-06	3.23	3.66	-0.43
cg13985437	LRRC32	8.8E-10	3.62E-06	22.33	24.27	-1.93
cg11071448	SYT2	9.51E-10	3.87E-06	39.04	41.57	-2.53
cg16391678	ITGAL	1.39E-09	5.61E-06	50.75	53.63	-2.88
cg16047567	DHRS3	1.44E-09	5.73E-06	25.22	27.38	-2.16
cg01899089	AHRR	1.59E-09	6.28E-06	44.79	47.07	-2.28
cg20886049	TSKU	1.69E-09	6.63E-06	65.01	67.43	-2.42
cg19427338		1.73E-09	6.71E-06	73.73	71.1	2.63
cg04018738	VARS	1.85E-09	7.1E-06	77.04	72.52	4.52
cg05603985	SKI	2.04E-09	7.77E-06	22.99	24.42	-1.43
cg19589396		2.07E-09	7.82E-06	55.16	58.07	-2.90
cg24540678		2.16E-09	8.07E-06	15.6	16.76	-1.16
cg13668129	HNRNPUL1	2.37E-09	8.79E-06	18.85	19.97	-1.12
cg26963277	KCNQ1OT1	3.25E-09	1.19E-05	82.42	85.17	-2.75
cg20244340	SLC24A3	3.33E-09	1.21E-05	22.55	24.99	-2.44
cg19940644		3.36E-09	1.21E-05	27.21	30.21	-3.00
cg23351584	PRSS23	3.88E-09	1.39E-05	9.46	10.33	-0.86
cg19713851	ALPP	5.44E-09	1.93E-05	30.96	36.28	-5.33
cg13916835	SMG6	6.04E-09	2.12E-05	63.02	66.12	-3.11
cg19614811	GPR15	6.22E-09	2.16E-05	72.39	77.93	-5.54
cg26118759	CD58	7.27E-09	2.51E-05	3.44	3.98	-0.54
cg07251887	LOC100130	7.41E-09	2.54E-05	38.55	40.62	-2.07
cg13038618		8.01E-09	2.72E-05	44.72	46.77	-2.05
cg06007201	FAM38A	8.29E-09	2.79E-05	5.75	6.5	-0.76
cg03604011	AHRR	9.29E-09	3.10E-5	4.17	3.37	0.80
cg09022230	TNRC18	9.36E-09	3.10E-5	61.93	64.42	-2.49
cg21446172	CAPN8	9.58E-09	3.15E-05	67.25	69.46	-2.21
cg08972170	C7orf41	9.69E-09	3.16E-05	46.29	43.2	3.09
cg26856289	SFRS13A	9.81E-09	3.17E-05	23.22	24.75	-1.52
cg17372101	CNTNAP2	1.16E-08	3.74E-05	44.87	42.25	2.62
cg21188533	CACNA1D	1.24E-08	3.96E-05	49.46	42.5	6.96
cg15693572		1.4E-08	4.43E-05	55.14	49.22	5.92
cg13633560	LRRC32	1.59E-08	4.99E-05	31.64	33.77	-2.13
cg24134897	TSPAN4	1.64E-08	5.10E-5	70.6	64.81	5.79
cg11557553	AHRR	1.72E-08	5.32E-05	75.93	74.37	1.56
cg26707709	SNED1	1.78E-08	5.45E-05	10.31	7.76	2.55
cg23126342	PCDH9	1.85E-08	5.64E-05	45.46	40.89	4.58
cg05875421	GPR68	1.97E-08	5.96E-05	9.83	10.87	-1.04
cg10814005	GPR68	2E-08	5.97E-05	9.79	11.09	-1.31
cg06171420		2.02E-08	5.97E-05	27.34	28.86	-1.52
cg24838345	MTSS1	2.02E-08	5.97E-05	63.45	67.25	-3.8
cg13185177	GP5	2.12E-08	6.23E-05	41.87	38.93	2.94
cg26718213	SNED1	2.24E-08	6.54E-05	23.27	18.44	4.83

cg12423733	MAS1L	2.68E-08	7.75E-05	14.25	12.26	1.98
cg26764244	GNG12	2.82E-08	8.11E-05	14.15	16.27	-2.12
cg11231349	NOS1AP	2.92E-08	8.33E-05	63.18	66.66	-3.49
cg06106428	ARHGAP20	3.06E-08	8.68E-05	46.3	43.32	2.98
cg09662411	GFI1	3.17E-08	8.92E-05	60.31	63.76	-3.44
cg04517044	SMARCD3	3.35E-08	9.34E-05	51.48	54.09	-2.61
cg05284742	ITPK1	3.36E-08	9.34E-05	60.72	62.62	-1.90
cg13127741	COMMD7	3.39E-08	9.35E-05	40.85	42.87	-2.02
cg08595501	IQGAP2	3.88E-08	1.06E-04	55.64	58.65	-3.01
cg25560398	ECEL1P2	3.89E-08	1.06E-04	55.99	57.58	-1.59
cg00336149	CACNA1D	4.31E-08	1.16E-04	32.85	29.51	3.34
cg07986378	ETV6	4.44E-08	1.19E-4	42.21	45.49	-3.29
cg13500388	CBFB	4.7E-08	1.25E-04	44.21	46.13	-1.93
cg16219322	AHRR	4.7E-08	1.25E-04	77.9	79.16	-1.26
cg01442064	EVC	4.89E-08	1.28E-04	22.4	23.95	-1.55
cg06648759		4.89E-08	1.28E-04	51.91	49.81	2.10
cg12729894	HCCA2(MOB2)	5.32E-08	1.38E-04	69.74	71.4	-1.66
cg27467282		5.38E-08	1.39E-04	76.55	74.59	1.96
cg04761231	RPL35	5.53E-08	1.42E-04	23.39	24.6	-1.21
cg13641317		5.86E-08	1.49E-04	21.15	19.24	1.91
cg07178945	FGF23	5.95E-08	1.51E-04	33.05	30.94	2.11
cg07151117	DUSP4	6.21E-08	1.57E-04	11.58	12.45	-0.87
cg15022400	TRIM69	6.36E-08	1.58E-04	13.76	15.23	-1.47
cg07465627	STXBP4	6.6E-08	1.63E-04	29.02	30.7	-1.68
ch.1.839062R	RUNX3	7.01E-08	1.73E-04	4.14	4.66	-0.53
cg26242531	ZFYVE21	7.41E-08	1.81E-04	35.97	34.11	1.85
cg18660898	CDC42SE1	7.54E-08	1.83E-04	7.01	7.9	-0.89
cg03188382	ALPP	7.61E-08	1.84E-04	35.86	37.85	-1.99
cg14569771		7.75E-08	1.86E-04	44.42	42.06	2.36
cg04368724	VARS	8.04E-08	1.92E-04	73.8	71.11	2.69
cg00501876	CSRNP1	8.94E-08	2.12E-04	54.5	56.49	-2.00
cg05302489	VARS	8.98E-08	2.12E-04	70.5	67.52	2.98
cg14420519		9.12E-08	2.14E-04	71.95	73.43	-1.48
cg07123182	KCNQ1OT1	9.21E-08	2.15E-04	80.86	84.22	-3.36
cg23657179	C10orf41	9.38E-08	2.18E-04	38.58	43.29	-4.71
cg21618017	RILPL1	9.55E-08	2.20E-04	5.38	5.83	-0.45
cg13787850		9.7E-08	2.23E-04	35.24	37.5	-2.26
cg10919522	C14orf43	1.01E-07	2.31E-04	17.88	19.6	-1.72
cg16398761	C14orf43	1.06E-07	2.42E-04	1.86	2.19	-0.34
cg25004427	AHRR	1.09E-07	2.45E-04	78.74	76.76	1.99
cg22403782	ALPP	1.09E-07	2.45E-04	27.33	30.42	-3.09
cg05969150	ZFHX3	1.11E-07	2.46E-04	34.95	33.58	1.37
cg16519923	ITGAL	1.21E-07	2.66E-04	56.07	58.45	-2.38
cg14901243		1.21E-07	2.66E-04	30.42	32.46	-2.02
cg04424621	HIST1H2BJ	1.24E-07	0.000271	9.38	10.46	-1.08
cg15638414		1.27E-07	0.000276	11.65	12.49	-0.84
cg05194346		1.28E-07	0.000277	49.26	46.27	3.00
cg12547807		1.29E-07	0.000278	25.98	27.39	-1.41
cg01340312	TUBB	1.3E-07	0.000278	5.19	5.71	-0.52

cg21854952	HIC1	1.33E-07	0.000284	19.95	18.27	1.68
cg00485194	ZNF385D	1.45E-07	0.000306	10.55	12.12	-1.57
cg11649376	ACSS3	1.45E-07	0.000306	47.39	49.18	-1.79
cg02583484	HNRNPA1	1.46E-07	0.000306	26.27	27.89	-1.62
cg02743070	ZMIZ1	1.51E-07	0.000314	67.26	68.81	-1.55
cg15878619	TUBB	1.61E-07	0.000334	6.17	6.74	-0.57
cg26221105	CAPN2	1.62E-07	0.000334	5.64	6.06	-0.43
cg17924476	AHRR	1.7E-07	0.000349	40.62	37.68	2.94
cg00569896		1.76E-07	0.000359	49.69	44.36	5.32
cg26844633		1.76E-07	0.000359	48.82	52.12	-3.31
cg22160883	MATR3	1.79E-07	0.000363	15.15	16.24	-1.08
cg19713429	CAPZB	2.01E-07	0.000404	18.86	20.56	-1.71
cg17024919	ZNF385D	2.02E-07	0.000404	26.47	30.52	-4.05
cg11621113	MORG1	2.08E-07	0.000416	25.89	27.27	-1.38
cg13784312	RAPGEF1	2.1E-07	0.000417	3.44	4.01	-0.57
cg25730428	MAS1L	2.14E-07	0.000423	28.32	26.65	1.67
cg23677833	AP1M1	2.3E-07	0.000451	8.99	9.89	-0.91
cg14977938	ZFYVE21	2.53E-07	0.000496	66.72	64.51	2.21
cg19325791		2.56E-07	0.000498	55.31	53.06	2.25
cg25305703		2.63E-07	0.000509	51.74	55.08	-3.34
cg10420527	LRP5	2.69E-07	0.000519	43.27	44.66	-1.40
cg11735008	HIVEP3	2.72E-07	0.000523	57.45	53.65	3.80
cg10788371	LRRC32	2.77E-07	0.000529	48.01	50.19	-2.18
cg02871659	SNHG9	2.8E-07	0.000533	21.51	24.16	-2.66
cg00210249	HK1	3.13E-07	0.000592	64.36	61.45	2.91
cg04945608	TTPAL	3.16E-07	0.000597	64.57	66.42	-1.85
cg25691553	NDUFAF3	3.23E-07	0.000605	3.72	4.16	-0.44
cg15644324	HIVEP3	3.25E-07	0.000607	51.20	45.51	5.69
cg24033122	ITGAL	3.27E-07	0.000607	14.63	16.77	-2.14
cg01963224		3.46E-07	0.000638	12.27	13.15	-0.89
cg12873476		3.46E-07	0.000638	59.46	62.17	-2.71
cg22644321	TRIB1	3.56E-07	0.000653	10.10	11.03	-0.93
cg13518625		3.61E-07	0.000659	4.95	5.84	-0.89
cg15417641	CACNA1D	3.83E-07	0.000696	50.76	45.40	5.36
cg14270346	SHB	3.84E-07	0.000696	75.60	77.53	-1.93
cg19925780		3.92E-07	0.000706	58.97	55.76	3.21
cg19089328	NADSYN1	3.95E-07	0.000708	68.14	65.35	2.79
cg18625627	TSHR	4.03E-07	0.00072	18.07	16.27	1.79
cg20716932	COMMD7	4.23E-07	0.000749	27.08	28.71	-1.64
cg16556677	KCNQ1OT1	4.25E-07	0.000749	69.81	71.72	-1.90
cg04641860	HNRNPF	4.27E-07	0.00075	57.88	60.29	-2.41
cg03292675	EPB49	4.35E-07	0.00076	25.22	27.15	-1.94
cg07525202	RHBDL3	4.46E-07	0.000775	71.22	73.43	-2.21
cg08553327	TNF	4.48E-07	0.000775	12.23	13.55	-1.32
cg23594345		4.49E-07	0.000775	33.91	37.49	-3.58
cg24563804	ACOT7	4.62E-07	0.000792	15.84	16.78	-0.94
cg10509323		4.64E-07	0.000792	6.53	7.23	-0.70
cg01406381	SLC1A5	4.65E-07	0.000792	4.86	5.53	-0.68
cg03254067	CLNK	4.67E-07	0.000793	8.00	7.38	0.62

cg20459687	STK24	4.69E-07	0.000793	2.52	2.87	-0.35
cg02519286	GAPDH	4.85E-07	0.000816	13.44	14.36	-0.91
cg23090529		4.97E-07	0.000834	28.95	31.61	-2.66
cg13525276	TSHR	5.04E-07	0.000843	23.71	21.59	2.12
cg17877600		5.16E-07	0.000859	45.08	46.95	-1.87
cg04716530	ITGAL	5.21E-07	0.000863	70.77	72.92	-2.15
cg19918734	ME3	5.38E-07	0.000886	9.77	10.53	-0.76
cg20005742		5.39E-07	0.000886	68.23	64.15	4.07
cg22635676	SNED1	5.59E-07	0.000915	12.95	7.80	5.15
cg11197630		5.63E-07	0.000918	67.93	70.60	-2.68
cg19713209	BRD2	5.84E-07	0.000949	1.82	2.04	-0.22
cg04678936	TP53BP1	5.93E-07	0.000957	23.45	24.68	-1.23
cg06214925	LOC641518	5.94E-07	0.000957	12.50	13.22	-0.72
cg03037030	TNF	6.08E-07	0.000973	2.84	3.21	-0.37
cg11436113		6.08E-07	0.000973	46.90	48.76	-1.86
cg10062919	RARA	6.12E-07	0.000974	39.72	41.08	-1.36
cg26729380	TNF	6.21E-07	0.000984	6.95	7.84	-0.89
cg11645155	SLC1A5	6.22E-07	0.000984	3.16	3.49	-0.33
cg25491122	PCDH9	6.26E-07	0.000986	73.39	70.13	3.27
cg04463638	CLDN5	6.32E-07	0.000991	66.96	68.79	-1.83
cg27449150		6.37E-07	0.000996	20.17	21.50	-1.32
cg14179389	GFI1	6.4E-07	0.000997	11.13	13.26	-2.14
cg05329352	ADRA2A	6.49E-07	0.001006	42.86	46.11	-3.25
cg26099045		6.54E-07	0.00101	50.60	47.75	2.85
cg22384356	FAM83A	6.57E-07	0.001012	49.63	45.45	4.18
cg22441770	CRTC2	7.17E-07	0.0011	57.58	59.09	-1.51
cg03139435	AURKA	7.23E-07	0.001105	15.41	16.68	-1.27
cg19583819	NRG2	7.39E-07	0.001125	10.66	11.86	-1.20
cg16547579	SLC23A2	7.54E-07	0.001143	13.65	14.88	-1.23
cg01904243	C14orf43	7.7E-07	0.00116	13.50	14.58	-1.09
cg08331398	PSMB8	7.7E-07	0.00116	68.70	70.49	-1.79
cg19956914	SUMF2	7.87E-07	0.00118	49.69	46.20	3.49
cg00871610	MIR802	8.23E-07	0.001229	45.42	47.54	-2.12
cg10590964		8.33E-07	0.00124	57.02	59.33	-2.31
cg08726900	ANKRD11	8.48E-07	0.001258	16.22	14.47	1.75
cg03359362	SLC1A5	8.64E-07	0.001277	2.87	3.22	-0.35
cg25512107	RPTOR	8.67E-07	0.001277	96.34	98.01	-1.68
cg09837977	LRRN3	8.89E-07	0.001305	74.31	75.98	-1.67
cg25748521	HIPK2	9.03E-07	0.001321	68.11	69.72	-1.61
cg03234777	AMICA1	1.00E-06	0.001455	10.47	11.59	-1.12
cg14989202	C1orf200	1.00E-06	0.001455	10.81	11.85	-1.04
cg08871244	FAM38A	1.02E-06	0.001482	8.04	8.97	-0.92
cg16290996	GAS5	1.03E-06	0.001487	18.23	19.74	-1.51
cg00925244		1.04E-06	0.001488	82.05	83.41	-1.36
cg11066209	FTSJ2	1.04E-06	0.001488	13.86	14.83	-0.97
cg04517079	FOXP4	1.05E-06	0.0015	56.32	58.26	-1.95
cg01207684	ADCY9	1.06E-06	0.0015	61.60	65.46	-3.87
cg23667432	ALPP	1.06E-06	0.0015	60.14	61.66	-1.52
cg26572392	KIAA1217	1.07E-06	0.001515	46.74	44.52	2.22

cg00024404	SERINC5	1.08E-06	0.001515	8.01	8.86	-0.84
cg19254163	GPR44	1.09E-06	0.001524	54.03	55.75	-1.71
cg12147622		1.11E-06	0.001546	44.32	46.37	-2.05
cg07166409	SEMA4C	1.15E-06	0.001604	3.99	4.39	-0.40
cg02730804	GRAMD3	1.16E-06	0.001606	66.91	65.14	1.77
cg02228160	HMHB1	1.17E-06	0.001615	27.72	25.95	1.77
cg06442294		1.17E-06	0.001615	11.06	12.05	-0.99
cg18405341	ATF4	1.17E-06	0.001615	46.41	47.86	-1.45
cg07180646	TMEM51	1.2E-06	0.001638	46.98	49.62	-2.64
cg01432620	ZIC2	1.23E-06	0.00167	7.78	8.43	-0.65
cg14093127		1.23E-06	0.00167	70.11	73.00	-2.90
cg23327334	GRK6	1.23E-06	0.00167	21.23	22.11	-0.88
cg13976502	C14orf43	1.27E-06	0.001711	42.16	43.78	-1.62
cg18450254	PRICKLE2	1.29E-06	0.001736	24.94	23.12	1.81
cg21140898		1.32E-06	0.001773	24.30	27.01	-2.70
cg04793090	CNBP	1.34E-06	0.001793	9.95	10.54	-0.58
cg01435643	MCF2L	1.35E-06	0.001793	43.11	39.68	3.43
cg25537245		1.35E-06	0.001793	8.72	9.36	-0.65
cg10389771	BCAN	1.39E-06	0.001845	28.30	30.13	-1.84
cg24420089	PTDSS2	1.4E-06	0.001853	35.36	33.47	1.89
cg11185549	MAP1LC3B2	1.41E-06	0.001853	9.76	10.59	-0.83
cg15187398	MOBKL2A	1.42E-06	0.001867	34.31	36.36	-2.05
cg13488013		1.44E-06	0.001883	22.36	23.58	-1.22
cg03785076	SNED1	1.46E-06	0.001899	59.41	60.97	-1.57
cg12165685	SLC1A5	1.46E-06	0.001899	4.53	5.12	-0.59
cg10843276	PCGF3	1.47E-06	0.0019	52.08	48.84	3.24
cg18642234	GPX1	1.54E-06	0.001979	43.91	45.42	-1.51
cg14120703	NOTCH1	1.56E-06	0.00201	49.86	51.22	-1.36
cg21878650	ADAMTS6	1.57E-06	0.002013	13.96	12.14	1.82
cg20751345		1.61E-06	0.00206	63.59	61.56	2.03
cg02637282		1.64E-06	0.002084	15.20	13.72	1.48
cg23230929		1.65E-06	0.002091	38.79	37.25	1.53
cg14675361	LMO7	1.66E-06	0.002097	17.75	16.11	1.64
cg14796406	SEMA3C	1.68E-06	0.002114	14.09	16.40	-2.32
cg26132737	ANKRD44	1.69E-06	0.00212	58.60	61.89	-3.30
cg01594685	IL32	1.73E-06	0.002156	69.02	70.78	-1.76
cg06819357	TECPR2	1.73E-06	0.002156	61.23	58.95	2.27
cg19719391		1.73E-06	0.002156	53.66	51.82	1.84
cg17412005	MUTYH	1.74E-06	0.002163	18.30	19.50	-1.21
cg14165660		1.79E-06	0.002212	70.10	68.37	1.73
cg01208318		1.81E-06	0.002235	30.69	33.71	-3.02
cg19783015		1.83E-06	0.002246	12.75	13.36	-0.61
cg14580211	C5orf62	1.84E-06	0.002262	61.78	63.85	-2.07
cg15937073	HIVEP3	1.86E-06	0.002278	27.34	24.38	2.96
cg21756476	RORA	1.89E-06	0.002301	49.43	47.33	2.10
cg26140475		1.91E-06	0.002319	14.78	15.89	-1.11
cg26529655	AHRR	1.95E-06	0.002362	64.69	66.35	-1.66
cg01763916	SMAP2	1.99E-06	0.002404	29.88	31.42	-1.54
cg05339037		2.04E-06	0.002455	41.67	43.15	-1.48

cg01127300		2.07E-06	0.002487	43.98	46.59	-2.61
cg00202479	VARS	2.1E-06	0.002522	76.75	75.10	1.65
cg04387396	SGPL1	2.22E-06	0.002649	2.66	2.96	-0.30
cg21766592	SLC1A5	2.26E-06	0.002689	6.29	7.12	-0.83
cg15874302		2.27E-06	0.002692	14.54	15.43	-0.89
cg07090714		2.31E-06	0.002737	27.51	29.36	-1.85
cg17953136	LAPTM4A	2.32E-06	0.002737	46.69	44.77	1.92
cg23110422	ETS2	2.36E-06	0.002779	67.83	70.50	-2.66
cg01882991		2.44E-06	0.002862	52.23	54.03	-1.80
cg17025683	SNORD78	2.45E-06	0.002871	10.43	11.57	-1.13
cg27004870	FAM38A	2.51E-06	0.002929	8.49	9.27	-0.78
cg04956244	RARA	2.54E-06	0.002956	59.50	58.21	1.29
cg27527798	ELFN1	2.56E-06	0.002974	65.62	67.87	-2.25
cg10482632	CSRP1	2.57E-06	0.002974	6.51	7.09	-0.57
cg25037384	OTX1	2.58E-06	0.002974	5.60	6.24	-0.65
cg23781467	VARS	2.63E-06	0.003031	57.24	55.49	1.75
cg07052041		2.69E-06	0.003094	7.17	7.84	-0.66
cg16254309	CNTNAP2	2.72E-06	0.003111	8.49	9.31	-0.82
cg00668559	NFKBIE	2.74E-06	0.003117	10.10	11.22	-1.12
cg02610360	TMEM136	2.75E-06	0.003117	36.00	33.07	2.93
cg18396403	TMEM184B	2.76E-06	0.003117	18.00	18.96	-0.95
cg23348010	SLC5A10	2.76E-06	0.003117	47.94	49.67	-1.73
cg22544881	FLJ43663	2.78E-06	0.003126	19.68	20.79	-1.12
cg19825437		2.81E-06	0.003153	67.52	69.22	-1.70
cg10581837	LMO7	2.83E-06	0.003167	30.06	28.42	1.64
cg13049261	SETD3	2.84E-06	0.003173	74.13	75.59	-1.46
cg14208102	TREX1	2.87E-06	0.003194	11.26	12.01	-0.75
cg03355101		2.9E-06	0.003212	4.17	4.61	-0.44
cg09701700	MIR146B	2.91E-06	0.003212	30.40	32.32	-1.92
cg19838043	ZFYVE21	2.91E-06	0.003212	57.66	55.50	2.15
cg16408970	BAMBI	2.92E-06	0.003214	7.77	8.42	-0.65
cg08335767	HMGA1	2.94E-06	0.003232	12.77	13.64	-0.87
cg25913882	CUBN	2.96E-06	0.003246	69.02	70.66	-1.64
cg24556382	GALNT7	3.04E-06	0.00332	60.51	63.39	-2.88
cg03699074	FAM38A	3.05E-06	0.003321	13.54	14.82	-1.28
cg08483768		3.05E-06	0.00332	44.75	40.18	4.57
cg11978634	ENOX1	3.12E-06	0.003381	68.73	66.41	2.32
cg06946797		3.17E-06	0.003428	17.59	19.22	-1.63
cg24851181	C3orf72	3.22E-06	0.003467	15.49	16.61	-1.11
cg00690082	STAT5A	3.23E-06	0.003467	23.87	24.79	-0.92
cg12877056	HDAC4	3.23E-06	0.003467	80.02	81.62	-1.60
cg00116430		3.29E-06	0.003525	44.96	47.39	-2.44
cg06595162	NCRNA0011	3.36E-06	0.003589	67.38	68.91	-1.53
cg23621097	HIC1	3.41E-06	0.003629	9.31	7.99	1.32
cg15636519	STAT4	3.43E-06	0.003633	8.13	9.08	-0.95
cg26898567	TMEM220	3.43E-06	0.003633	68.53	70.38	-1.86
cg00464814	ATXN1	3.46E-06	0.003659	13.66	14.61	-0.94
cg13937905	RARG	3.48E-06	0.003667	82.21	85.12	-2.90
cg07285983	RABGAP1L	3.49E-06	0.003668	75.73	73.27	2.46

cg02793162	CORO2B	3.51E-06	0.003668	38.02	36.37	1.65
cg16969872	RBM26	3.51E-06	0.003668	61.35	63.63	-2.27
cg15482893	BSDC1	3.52E-06	0.003668	21.33	20.00	1.34
cg23480341	SLPI	3.56E-06	0.003707	6.69	7.52	-0.83
cg14897188	RNASEK	3.59E-06	0.003725	59.03	60.60	-1.57
cg01538731	KLHDC4	3.6E-06	0.003725	59.14	54.63	4.51
cg16771652	NOD2	3.65E-06	0.003769	19.14	20.16	-1.02
cg23279756		3.66E-06	0.003769	16.03	14.66	1.36
cg02767093	STK24	3.71E-06	0.003808	24.42	25.42	-0.99
cg02254407	PLEKHB1	3.73E-06	0.003825	77.52	79.62	-2.09
cg15331996	SPOCK2	3.74E-06	0.003825	23.36	24.56	-1.20
cg05830220	KLHDC4	3.81E-06	0.00388	70.97	67.28	3.69
cg15380836	RILP	3.81E-06	0.00388	9.50	10.09	-0.59
cg26128192		3.86E-06	0.003919	12.05	12.86	-0.80
cg05438378	SMAD3	3.95E-06	0.003994	18.86	20.67	-1.81
cg03935116	FAM60A	4.00E-06	0.004034	11.24	12.13	-0.89
cg10717214	TNF	4.06E-06	0.004089	7.71	8.42	-0.71
cg05661533	LPCAT1	4.11E-06	0.00412	30.53	32.21	-1.68
cg18966401	ELF3	4.11E-06	0.00412	59.98	61.53	-1.55
cg08822075	NFE2L3	4.22E-06	0.004217	14.04	16.32	-2.28
cg03474926	RALGDS	4.29E-06	0.004269	31.04	32.13	-1.09
cg26937798	IL4R	4.3E-06	0.004269	2.72	3.10	-0.38
cg02385153	AHRR	4.31E-06	0.004269	79.44	77.76	1.67
cg11412468		4.31E-06	0.004269	45.93	44.03	1.90
cg21913886	TMEM51	4.32E-06	0.004269	69.23	71.68	-2.45
cg15519474	PRKCA	4.45E-06	0.004382	81.46	82.94	-1.48
cg03440944	C7orf40	4.48E-06	0.004397	61.04	62.76	-1.72
cg06216408		4.48E-06	0.004397	25.76	26.98	-1.23
cg21222743	TNF	4.51E-06	0.004418	5.51	6.16	-0.65
cg20698421	SLC1A4	4.56E-06	0.00444	35.98	39.94	-3.96
cg25607920	HIVEP3	4.56E-06	0.00444	34.48	28.97	5.52
cg15975802	PTPN6	4.59E-06	0.004455	17.28	18.09	-0.81
cg02832697	TRIM2	4.66E-06	0.004506	35.76	37.39	-1.64
cg03630015	WNT10A	4.66E-06	0.004506	66.97	69.00	-2.03
cg10951873	RUNX3	4.7E-06	0.004531	5.97	6.50	-0.53
cg00498211	SLC1A4	4.77E-06	0.004593	18.25	20.77	-2.52
cg19903071	BAZ2B	4.85E-06	0.004631	21.14	19.79	1.35
cg14550518	ZNF385D	4.86E-06	0.004631	3.75	4.41	-0.66
cg17098415		4.86E-06	0.004631	2.04	2.28	-0.23
cg17320698	MPPED2	4.88E-06	0.00464	55.18	57.43	-2.25
cg24230340	HTRA2	4.92E-06	0.004666	12.24	13.53	-1.29
cg26305174	SLC12A9	4.93E-06	0.004666	36.72	34.78	1.93
cg20700731	EDN2	4.94E-06	0.004669	48.67	50.08	-1.41
cg00942495	FBXO22	4.96E-06	0.004673	86.35	84.06	2.29
cg15428620	SFXN3	5.04E-06	0.004736	31.11	32.24	-1.13
cg25446789	DTNB	5.11E-06	0.004797	41.12	42.92	-1.81
cg04231319	MLLT10	5.19E-06	0.004856	23.23	24.30	-1.07
cg19514721		5.28E-06	0.004933	56.63	54.69	1.94
cg26356892	SLC25A17	5.36E-06	0.004981	3.11	2.50	0.61

cg03345925	ZC3H3	5.42E-06	0.005032	65.50	63.62	1.88
cg00640314	SNORD87	5.49E-06	0.005064	63.85	65.98	-2.13
cg14012925	CUX1	5.5E-06	0.005064	37.52	35.33	2.18
cg06830167	CAMTA1	5.54E-06	0.005097	15.97	17.15	-1.18
cg25686812	SEC22C	5.59E-06	0.005126	6.35	6.92	-0.57
cg26343958		5.63E-06	0.00515	17.66	19.19	-1.52
cg22383924	TP73	5.74E-06	0.005247	51.56	53.24	-1.68
cg15058210	HDAC4	5.83E-06	0.005311	10.81	11.94	-1.13
cg00741986	TNIP2	5.94E-06	0.005394	46.20	47.85	-1.65
cg14011327		5.94E-06	0.005394	8.13	8.82	-0.69
cg24389054	HOXA7	5.98E-06	0.005411	68.67	70.93	-2.26
cg05267427	ZNF827	6.05E-06	0.005468	80.54	76.69	3.85
cg02917867		6.12E-06	0.005518	28.72	30.03	-1.32
cg16178855	PMEPA1	6.17E-06	0.005539	72.88	71.53	1.34
cg21436456	ZNF385D	6.17E-06	0.005539	5.38	6.16	-0.79
cg00911794	HIC1	6.22E-06	0.005571	13.88	12.25	1.62
cg17001464	FLJ43663	6.24E-06	0.005571	79.49	80.84	-1.34
cg25223634	C10orf26	6.25E-06	0.005576	36.40	38.50	-2.10
cg10717312	SNED1	6.42E-06	0.005715	44.61	41.18	3.43
cg15903282	EPHB1	6.46E-06	0.005721	68.61	70.43	-1.82
cg26669717	PRKAR1B	6.46E-06	0.005721	50.71	52.28	-1.57
cg08384239	TANC1	6.58E-06	0.005816	33.98	32.67	1.31
cg26057969	TPRA1	6.61E-06	0.005829	15.26	16.01	-0.75
cg22998476		6.67E-06	0.005874	25.62	27.01	-1.40
cg25607249	SLC1A5	6.7E-06	0.005882	6.85	7.57	-0.72
cg26077378	ZNF385D	6.77E-06	0.005929	11.60	13.32	-1.72
cg12611488	SKI	6.79E-06	0.005934	80.77	77.61	3.16
cg16509061	TERT	6.83E-06	0.00596	78.84	79.98	-1.14
cg26542660	CEP135	7.09E-06	0.00617	11.87	12.65	-0.79
cg26504421	SDK2	7.13E-06	0.006195	48.49	53.81	-5.33
cg05593667		7.2E-06	0.006218	14.78	16.09	-1.31
cg14064762	TRAF1	7.2E-06	0.006218	9.66	10.57	-0.90
cg15471946	BRWD1	7.2E-06	0.006218	5.41	5.89	-0.48
cg20267775		7.25E-06	0.006251	75.22	76.43	-1.22
cg20187173		7.29E-06	0.006268	75.67	73.73	1.94
cg23222488		7.33E-06	0.006288	63.75	61.32	2.42
cg05655806	CD96	7.42E-06	0.006342	38.28	41.06	-2.78
cg05616472	EHMT1	7.43E-06	0.006342	11.11	11.79	-0.68
cg01873977	MTSS1	7.44E-06	0.006342	59.33	61.69	-2.36
cg11229399		7.49E-06	0.006374	8.13	7.30	0.83
cg13238479	MAB21L1	7.53E-06	0.006393	28.59	30.11	-1.52
cg16736826	EDN2	7.6E-06	0.006439	33.20	34.44	-1.24
cg11556164	LRRN3	7.61E-06	0.006439	64.27	66.86	-2.59
cg19593285	E2F1	7.76E-06	0.006551	63.25	65.94	-2.69
cg20465954	SOAT2	7.8E-06	0.006572	3.74	4.09	-0.35
cg02736908		7.88E-06	0.006621	58.15	61.01	-2.85
cg04942107		7.92E-06	0.006643	69.95	71.40	-1.44
cg20309891	MEOX1	7.95E-06	0.006651	58.22	59.64	-1.42
cg09496748	ANKRD44	8.03E-06	0.006704	72.52	74.68	-2.16

cg07500325		8.05E-06	0.006709	44.70	47.36	-2.66
cg19197419	UBE2C	8.1E-06	0.006742	11.44	12.60	-1.16
cg05901447	SLC34A1	8.13E-06	0.006748	76.17	77.29	-1.12
cg18325389		8.15E-06	0.006756	24.71	26.17	-1.46
cg26170244		8.37E-06	0.00692	16.24	17.39	-1.16
cg17318719	TMPRSS4	8.45E-06	0.006972	29.05	30.16	-1.11
cg22417733	FBXO5	8.57E-06	0.007062	15.12	16.75	-1.63
cg13608545		8.72E-06	0.007153	55.23	57.41	-2.19
cg16134718		8.72E-06	0.007153	65.61	67.14	-1.53
cg05222243	COL13A1	8.74E-06	0.007153	70.98	72.29	-1.31
cg21746120	LRP5	8.88E-06	0.007253	33.70	34.72	-1.03
cg01617071	ADAP1	8.9E-06	0.00726	46.59	49.12	-2.53
cg08738403	AHCYL2	9.00E-06	0.007324	4.19	4.58	-0.39
cg15963913	ZFYVE21	9.03E-06	0.007331	67.23	65.35	1.88
cg03433699	RNASEK	9.25E-06	0.007481	5.35	5.79	-0.44
cg19372602		9.47E-06	0.007646	33.96	35.84	-1.88
cg21552014		9.51E-06	0.007662	68.16	66.25	1.91
cg25838968	PLXNA2	9.55E-06	0.007676	44.71	46.52	-1.81
cg26276120	TPI1	9.56E-06	0.007676	15.53	16.63	-1.11
cg16744741	PRKG2	9.62E-06	0.007708	33.63	31.96	1.67
cg04137490	DOCK5	9.8E-06	0.007835	82.87	79.53	3.34
cg06500714	VENTX	9.98E-06	0.007961	1.56	1.30	0.26
cg19057830	C10orf41	0.00001	0.007981	58.86	61.59	-2.73
cg05673882	POLK	1.01E-05	0.008008	23.34	25.01	-1.66
cg25421530	SORBS1	1.01E-05	0.008016	74.47	72.35	2.11
cg26891494	ZNF365	1.01E-05	0.008011	79.59	77.46	2.14
cg03641640	RASA3	1.02E-05	0.008016	84.83	86.80	-1.97
cg10179300	TRIO	1.02E-05	0.008016	75.11	76.95	-1.84
cg16201146		1.02E-05	0.008016	63.49	65.13	-1.64
cg25799109	ARHGEF3	1.02E-05	0.008016	26.75	28.80	-2.05
ch.5.67026991F		1.04E-05	0.008151	1.51	1.25	0.26
cg15743533	FAM110A	1.06E-05	0.008283	11.28	12.13	-0.85
cg22437284	SRC	1.06E-05	0.008283	78.00	76.72	1.29
cg25420507	LGALS7	1.06E-05	0.008283	15.65	17.00	-1.35
cg01118348		1.07E-05	0.008298	59.36	60.74	-1.38
cg15775914	CHML	1.07E-05	0.008298	56.32	54.48	1.84
cg00687135	ZNF385D	1.08E-05	0.00836	36.34	39.05	-2.71
cg14920061		1.09E-05	0.008457	11.22	12.10	-0.88
cg09109520	GPR56	1.10E-05	0.008508	26.87	25.21	1.66
cg11693709	PAK6	1.10E-05	0.008482	36.59	34.55	2.04
cg13832372	LHX6	1.11E-05	0.008514	10.38	12.22	-1.84
cg15108334	RPS12	1.12E-05	0.008617	64.81	66.50	-1.69
cg15474579	CDKN1A	1.16E-05	0.008923	51.21	53.03	-1.82
cg25013095		1.18E-05	0.009014	86.66	88.89	-2.23
cg03253303	RAB11FIP4	1.19E-05	0.00908	55.63	57.43	-1.80
cg00816037	FAM38A	1.20E-05	0.009125	7.00	7.97	-0.97
cg26878655	RARA	1.20E-05	0.009163	65.34	66.94	-1.60
cg16983588	PRDM10	1.21E-05	0.009229	43.00	40.46	2.53
cg03814093	KIAA0922	1.22E-05	0.00923	12.24	13.80	-1.56

cg13184736	GNG12	1.24E-05	0.009376	26.41	29.78	-3.37
cg22188918	CLDN15	1.24E-05	0.009352	52.79	50.52	2.27
cg24688690	AHRR	1.24E-05	0.009376	60.17	61.56	-1.39
cg07068045		1.25E-05	0.00939	72.07	75.36	-3.29
cg16903811		1.25E-05	0.009408	16.10	17.22	-1.12
cg07993586	DNAJB6	1.26E-05	0.009455	69.07	70.69	-1.61
cg17417856	PRMT1	1.26E-05	0.009463	54.63	56.45	-1.82
cg02639359	FCHO1	1.29E-05	0.009597	29.13	30.79	-1.67
cg06298346	ANKRD33B	1.29E-05	0.009597	16.52	17.54	-1.02
cg17823346	ZMIZ1	1.29E-05	0.009597	41.49	43.33	-1.83
cg01651915		1.30E-05	0.009642	22.03	24.45	-2.41
cg20134287	AUTS2	1.30E-05	0.009652	13.25	14.49	-1.24
cg23637791	ODF3L1	1.30E-05	0.009642	13.22	14.00	-0.77
cg13583535	MBP	1.35E-05	0.009988	9.60	10.29	-0.70
cg13591783	ANXA1	1.36E-05	0.010007	11.85	12.72	-0.87
cg14371731	ZMIZ1	1.36E-05	0.010007	2.24	1.38	0.86
cg16783186		1.36E-05	0.010007	37.30	35.17	2.13
cg01979157	SKI	1.37E-05	0.010035	4.84	5.39	-0.55
cg10920316		1.37E-05	0.010028	7.36	8.00	-0.64
cg05713794	GP5	1.38E-05	0.010107	81.29	79.80	1.48
cg09338136	AHRR	1.39E-05	0.010175	17.48	18.76	-1.28
cg04970434	GP1BA	0.000014	0.010232	65.68	64.03	1.64
cg00449189		1.41E-05	0.010285	11.02	11.85	-0.83
cg11555067	INPP4A	1.46E-05	0.010612	23.71	25.10	-1.39
cg00917251	RCAN3	1.47E-05	0.010624	10.35	11.14	-0.79
cg17250929	S100A5	1.47E-05	0.010624	42.89	41.38	1.52
cg07452776	GPR68	1.49E-05	0.010726	6.98	7.38	-0.39
cg10650821	TNF	1.49E-05	0.010732	8.36	8.98	-0.62
cg07485775	NEURL1B	0.000015	0.010774	68.40	70.81	-2.41
cg21345826	APOBEC3A	0.000015	0.010774	75.84	74.50	1.34
cg03646605	ME3	1.51E-05	0.010805	78.20	79.75	-1.55
cg21241410		1.51E-05	0.010805	14.28	15.30	-1.02
cg21084508	CCDC108	1.52E-05	0.010856	43.57	40.50	3.07
cg22533683	MIR193A	1.52E-05	0.010858	48.94	51.82	-2.88
cg11209447	SCNN1A	1.54E-05	0.010951	69.21	70.48	-1.27
cg06872036	RASSF5	1.55E-05	0.010999	78.30	79.41	-1.11
cg26859016	IKBKE	1.58E-05	0.011225	65.84	67.44	-1.61
cg05091997		1.59E-05	0.011225	3.55	3.99	-0.44
cg09608073	CHSY3	1.59E-05	0.01125	8.58	9.09	-0.51
cg20322193	RALA	1.59E-05	0.011225	28.66	30.57	-1.91
cg18582010	ASAP1	0.000016	0.011285	65.04	63.01	2.03
cg00134210	FAM107B	1.61E-05	0.011341	3.21	3.59	-0.38
cg05726935	AKT1	1.62E-05	0.011379	54.25	55.56	-1.31
cg10666013	ISLR	1.62E-05	0.01135	77.55	78.56	-1.01
cg12614244	AP2A2	1.63E-05	0.011379	80.02	78.69	1.33
cg24497361	RHOG	1.63E-05	0.011379	27.47	28.72	-1.25
cg02755131	CDH13	1.65E-05	0.011528	74.50	72.53	1.97
cg07381806	MOBKL2A	1.66E-05	0.011528	21.45	23.60	-2.15
cg20850016	ARL8B	1.66E-05	0.011528	5.35	5.84	-0.49

cg20871826	CAMKK1	1.66E-05	0.011535	21.42	22.26	-0.83
cg01605984	SNORD24	1.67E-05	0.011551	61.23	62.58	-1.35
cg21840875	SMCR7L	1.67E-05	0.01154	48.73	50.03	-1.30
cg04149978	CASZ1	1.68E-05	0.011592	70.11	72.09	-1.98
cg12556164	GALNTL6	1.68E-05	0.011592	77.01	78.01	-1.00
cg05307957	ARID1A	1.70E-05	0.011681	3.05	3.35	-0.30
cg05460226	PIK3R5	1.70E-05	0.011681	31.79	33.36	-1.57
cg18252924	STK24	1.71E-05	0.011734	62.99	64.41	-1.41
cg18503679	ZNF385D	1.71E-05	0.011728	1.33	1.89	-0.56
ch.2.30415474F		1.72E-05	0.011734	3.41	3.72	-0.31
cg10865498	SRC	1.73E-05	0.011806	35.87	34.60	1.27
cg01510278		1.74E-05	0.011812	3.91	4.28	-0.37
cg25114611	FKBP5	1.74E-05	0.011812	31.00	32.12	-1.12
cg26282236	RAD52	1.74E-05	0.01183	53.81	51.24	2.57
cg14484649	FAM181B	1.75E-05	0.011874	16.15	16.85	-0.71
cg00357551	FAM196B	1.76E-05	0.01193	33.16	31.56	1.60
cg05903736	HDAC4	1.79E-05	0.012076	12.04	13.20	-1.15
cg22698028	AHRR	1.81E-05	0.012215	11.32	10.22	1.10
cg26572452		1.82E-05	0.012253	14.20	15.09	-0.89
cg16649560	IL4R	1.83E-05	0.012306	16.74	17.53	-0.79
ch.12.55659191R		1.85E-05	0.012456	1.82	2.02	-0.20
cg15505219	PRKCH	1.86E-05	0.012492	75.33	77.26	-1.94
cg00026474	ST3GAL1	1.87E-05	0.012504	19.36	20.70	-1.34
cg07741984	C3orf52	1.87E-05	0.012496	11.84	12.43	-0.59
cg25252598		1.89E-05	0.012637	6.05	6.50	-0.45
cg15576342		1.91E-05	0.012711	68.57	69.92	-1.36
cg12303084	ZMYND8	1.92E-05	0.012743	12.85	13.80	-0.95
cg24296397	BSN	1.93E-05	0.01278	48.77	47.14	1.63
cg15677364	TUBB	1.94E-05	0.012813	22.09	23.41	-1.32
cg20146909	LRRC8D	1.95E-05	0.0129	64.36	65.80	-1.44
cg26870865	TFDP1	1.96E-05	0.0129	69.34	67.93	1.40
cg23072383	SLC35E4	1.99E-05	0.013092	5.90	6.49	-0.59
cg06554062	GATAD2A	0.00002	0.013177	5.11	5.46	-0.35
cg00505001	MAB21L1	2.01E-05	0.013179	13.32	14.22	-0.90
cg00397859	C20orf7	2.02E-05	0.013237	30.80	28.47	2.33
cg17418085	LAPTM5	2.04E-05	0.013347	5.55	5.97	-0.42
cg18879828		2.04E-05	0.013347	68.62	69.80	-1.19
cg06580770	TNXB	2.05E-05	0.013373	66.02	67.35	-1.32
cg22638542	SEC22C	2.05E-05	0.013347	82.09	81.13	0.96
cg02610723	FAM38A	2.07E-05	0.013438	5.36	5.90	-0.54
cg24709951		2.07E-05	0.013438	40.32	37.08	3.25
cg01810186		2.09E-05	0.013524	43.55	46.41	-2.86
cg16190265	PRKAR1B	2.11E-05	0.013666	26.80	28.69	-1.89
cg09570614		2.15E-05	0.013888	42.49	41.00	1.49
cg24427243	TNFRSF8	2.15E-05	0.013868	55.20	57.36	-2.15
cg17328794	NRBP2	2.19E-05	0.014122	3.58	3.85	-0.26
cg10099304	TLE6	0.000022	0.014122	7.37	7.97	-0.60
cg19137417		0.000022	0.014146	73.21	75.13	-1.92
cg04372535	AP2A2	2.22E-05	0.014211	81.01	79.66	1.35

cg06184825	CPNE5	2.22E-05	0.014211	62.79	65.67	-2.88
cg18329753		2.25E-05	0.014379	77.86	79.50	-1.64
cg04258133	TNNT3	2.26E-05	0.01441	79.20	77.30	1.89
cg20124610	CARS2	2.26E-05	0.01441	45.32	46.71	-1.39
cg25341560	SCN1B	2.28E-05	0.014479	43.91	46.77	-2.86
cg03999941		2.30E-05	0.014578	62.01	64.51	-2.50
cg08416875	MAN1A1	2.30E-05	0.014578	5.78	5.41	0.37
cg11674448		2.30E-05	0.014578	63.09	60.66	2.43
cg01017464	SNORD58A	2.31E-05	0.014583	17.14	18.49	-1.34
cg00436663	LOC400927	2.32E-05	0.014669	4.87	5.23	-0.36
cg16151538	TRPC4AP	2.33E-05	0.014669	48.91	47.51	1.40
cg18369972	GAS7	2.34E-05	0.014706	18.92	19.98	-1.06
cg26296488	DRD5	2.34E-05	0.014719	8.05	6.86	1.19
cg03047995	FITM1	2.40E-05	0.015045	23.91	22.61	1.30
cg07207669	EFNA1	2.40E-05	0.015045	54.99	53.15	1.83
cg03469233		2.41E-05	0.015083	47.40	44.40	3.00
cg23233742		2.41E-05	0.015074	60.82	62.54	-1.72
cg05396397	NPPA	2.43E-05	0.015157	45.04	42.39	2.65
cg21108085	CD82	2.43E-05	0.015157	10.31	11.00	-0.69
cg05076755	LRRC32	2.45E-05	0.015241	77.95	79.44	-1.49
cg08202265	TAC1	2.46E-05	0.015241	87.87	85.29	2.58
cg20305005	SCN2A	2.49E-05	0.015414	59.95	58.02	1.92
cg11390978	SIN3B	2.51E-05	0.015492	69.70	73.30	-3.59
cg21559386	HNF4A	2.52E-05	0.015535	66.08	67.86	-1.78
cg09257526	IL6R	2.58E-05	0.01588	20.95	21.63	-0.69
cg26038582	HIVEP3	2.58E-05	0.015876	39.98	34.35	5.63
cg04471192		0.000026	0.015987	82.27	80.83	1.43
cg01377807	C3orf26	2.61E-05	0.015987	52.30	54.37	-2.07
cg07651540	LOC728606	2.61E-05	0.015987	54.55	52.85	1.70
cg07660627	ACACA	2.61E-05	0.015987	46.27	43.19	3.08
cg12231340	KRT81	2.68E-05	0.016339	74.83	77.32	-2.49
cg21086153	CDC42BPB	2.68E-05	0.016339	9.56	8.82	0.74
cg00496272	CENPT	2.69E-05	0.016364	10.16	10.85	-0.68
cg12983285	PTK6	2.69E-05	0.016364	6.53	7.23	-0.70
cg17769442	ZNF296	2.70E-05	0.016364	7.83	8.39	-0.57
cg20912205	NAT6	2.70E-05	0.016364	20.37	21.36	-0.99
cg09718582		2.74E-05	0.016568	54.93	57.37	-2.44
cg16660971	RPTOR	2.74E-05	0.016565	71.31	68.05	3.26
cg22851561	C14orf43	2.76E-05	0.016682	42.09	43.66	-1.57
cg14943908	BAT2	2.79E-05	0.016792	6.96	7.37	-0.41
cg07765674		2.81E-05	0.016922	56.36	57.66	-1.30
cg22510662	C5orf33	2.83E-05	0.017009	66.98	64.98	2.00
cg21916461	LRP5	2.85E-05	0.017103	13.19	14.09	-0.90
cg15763258	FLI1	2.87E-05	0.017159	68.48	69.92	-1.45
cg24149237		2.87E-05	0.017159	26.93	27.90	-0.97
cg19295068	GLTSCR1	2.89E-05	0.017262	23.57	21.71	1.86
cg02590572	CHI3L2	2.90E-05	0.017262	78.35	76.95	1.39
cg11788367	ACVRL1	2.90E-05	0.017262	82.11	83.03	-0.92
cg06338710	GFI1	2.94E-05	0.017435	77.15	79.05	-1.90

cg05529343		2.95E-05	0.017489	34.00	29.83	4.17
cg13411554	CACNA1D	2.99E-05	0.017702	32.64	27.77	4.87
cg22116041	TMEM154	3.01E-05	0.017821	14.04	14.85	-0.80
cg13074055		3.02E-05	0.017837	38.84	42.78	-3.94
cg06087019		3.03E-05	0.017889	9.12	9.69	-0.57
cg15410835		3.04E-05	0.017889	43.45	46.60	-3.15
cg27287951	ADAP1	3.05E-05	0.017889	86.29	84.05	2.24
cg22932220	NME2	3.07E-05	0.017991	3.52	3.79	-0.27
cg23762517	HIVEP3	3.07E-05	0.017991	39.19	35.08	4.10
cg13578306		3.08E-05	0.018008	71.76	70.28	1.48
cg02756939	C1orf97	3.11E-05	0.018127	13.90	15.57	-1.67
cg07218081	CLEC3B	3.12E-05	0.01818	62.86	64.39	-1.53
cg25141995	VDAC1	3.18E-05	0.018467	79.98	81.08	-1.10
cg11145461	CSRP1	3.19E-05	0.018491	73.65	75.45	-1.80
cg14295611		0.000032	0.018545	69.87	68.10	1.77
cg16360836	HDAC4	3.22E-05	0.018636	14.97	15.87	-0.90
cg24127989	IMPDH1	3.22E-05	0.018636	4.35	4.64	-0.30
cg05603910	ANO9	0.000033	0.019074	75.94	73.94	2.00
cg11014740		3.32E-05	0.019165	31.77	32.80	-1.03
cg18400079	AFAP1	3.37E-05	0.019373	68.87	66.64	2.23
cg26373411	WDR67	3.37E-05	0.019373	4.55	4.90	-0.35
cg16043888	TNFSF14	0.000034	0.019513	71.26	72.47	-1.21
cg07902156	LPCAT1	3.41E-05	0.019537	23.32	25.00	-1.68
cg23343486	SEPT7	3.42E-05	0.019578	4.77	5.18	-0.41
cg05557932	CREBBP	3.45E-05	0.019713	29.52	30.78	-1.26
cg02524834	CCND3	3.49E-05	0.019864	77.60	74.93	2.67
cg05906166	KLF2	3.49E-05	0.019864	24.53	27.48	-2.95
cg20017995	SNORD24	3.49E-05	0.019864	61.23	62.56	-1.33
cg01243823	NOD2	0.000035	0.01987	22.37	24.13	-1.75
cg02379082		0.000035	0.019877	8.63	9.24	-0.62
cg05875239		3.51E-05	0.019911	78.44	74.28	4.17
cg03044533	MAML2	3.54E-05	0.020002	36.77	35.03	1.74
cg04625615	ITPKA	3.54E-05	0.020002	72.40	74.22	-1.82
cg14232218		3.54E-05	0.020002	82.84	84.85	-2.01
cg21091547	CDKN1A	3.55E-05	0.020009	57.42	59.84	-2.42
cg21696256		3.55E-05	0.020009	80.03	81.49	-1.46
cg12406027	PAK4	3.56E-05	0.020015	20.95	21.93	-0.99
cg03743262		3.57E-05	0.020049	76.39	75.06	1.33
cg12198704		3.58E-05	0.020082	13.76	14.76	-1.00
cg02029908	DUSP1	3.59E-05	0.020118	86.31	84.83	1.48
cg13855261	HS3ST3B1	0.000036	0.020129	15.36	16.46	-1.10
cg23646614	PTPRJ	0.000036	0.020129	18.17	18.78	-0.61
cg13543915		3.61E-05	0.020137	63.24	64.49	-1.25
cg01657995	C6orf48	3.66E-05	0.020334	8.77	9.50	-0.74
cg23162201	LMO1	3.66E-05	0.020334	71.04	68.52	2.52
cg15095917	CD68	3.67E-05	0.020334	13.10	13.96	-0.85
cg26724967	IL32	3.67E-05	0.020334	60.29	61.94	-1.65
cg14408447		3.68E-05	0.020334	44.37	41.07	3.29
cg02003183	CDC42BPB	3.69E-05	0.020407	6.82	6.06	0.76

cg15429214		3.72E-05	0.02051	62.79	66.27	-3.48
cg19798735	IMMP2L	3.74E-05	0.020601	58.75	60.83	-2.08
cg01020987	C1orf174	3.75E-05	0.020621	22.09	23.54	-1.46
cg21698310	PPP1R9B	3.75E-05	0.020621	10.39	11.17	-0.78
cg20727233	ASNS	3.76E-05	0.020621	8.70	9.26	-0.56
cg23721083	ITGA11	3.83E-05	0.020968	5.92	6.44	-0.52
cg02032558		3.84E-05	0.020992	26.14	27.96	-1.82
cg25191725	PYY2	3.85E-05	0.021046	81.21	82.53	-1.32
cg05058354	INTS1	3.87E-05	0.021115	56.59	57.95	-1.35
cg27017172	POLR2C	3.87E-05	0.021115	7.19	7.84	-0.65
cg11832281	CUGBP2	3.94E-05	0.021434	4.00	4.34	-0.34
cg04652522	CANX	3.95E-05	0.021499	14.26	15.01	-0.75
cg03508212	RPS7	3.98E-05	0.021567	7.17	7.78	-0.61
cg17690322	CORO1B	3.98E-05	0.021564	58.49	59.89	-1.40
cg21171320	TRPM3	3.98E-05	0.021564	22.93	23.85	-0.92
cg03913271	MICALL1	4.00E-05	0.02162	4.29	4.53	-0.25
cg13434489	PRDM16	4.00E-05	0.02162	80.16	81.91	-1.75
cg00091589	KLHDC4	4.03E-05	0.02173	88.64	87.03	1.62
cg00148223		4.04E-05	0.021763	62.14	63.53	-1.39
cg06361984	NDE1	4.05E-05	0.021792	81.49	79.90	1.58
cg07177756	SNORD75	4.06E-05	0.021792	18.35	19.91	-1.56
cg24455969		4.06E-05	0.021792	46.35	49.19	-2.84
cg08772028	AMICA1	4.08E-05	0.02185	37.98	39.56	-1.58
cg20942162		4.01E-05	0.021945	46.37	44.97	1.39
cg27171474	CDCA8	4.11E-05	0.021951	85.85	84.87	0.98
cg08529529	ALOX5AP	4.15E-05	0.022118	10.55	11.29	-0.74
cg12392519	PHOSPHO1	4.15E-05	0.022118	43.95	41.81	2.14
cg22601421	RBM47	4.15E-05	0.022118	4.25	3.93	0.32
cg09081994	CDGAP	4.16E-05	0.022132	65.69	64.15	1.55
cg14099685	CUGBP1	4.21E-05	0.022358	26.38	27.35	-0.97
cg25462795	HNRNPL	4.22E-05	0.02238	4.21	4.50	-0.29
cg09646173	PDE6A	4.23E-05	0.022381	40.38	42.10	-1.71
cg00390253	SLC38A3	4.25E-05	0.022489	67.62	68.88	-1.27
cg05756780	IL6R	4.27E-05	0.022489	23.25	24.18	-0.93
cg06806711	MS4A1	4.28E-05	0.022489	35.85	34.67	1.18
cg09933323	CENPF	4.28E-05	0.022489	83.01	80.72	2.29
cg21927363	C9orf93	4.28E-05	0.022489	7.13	7.52	-0.39
cg26189021	KCNC4	4.28E-05	0.022489	58.42	59.95	-1.53
cg09325711	RALA	4.29E-05	0.022489	5.77	6.32	-0.55
cg11417675	UCP2	4.29E-05	0.022489	7.86	8.53	-0.67
cg05055821		4.31E-05	0.022573	9.82	9.18	0.64
cg24535439	MMP23B	4.33E-05	0.022616	19.71	21.25	-1.54
cg25568061		4.33E-05	0.022616	18.33	17.62	0.71
cg06803614	NT5C1A	4.34E-05	0.022624	70.07	71.39	-1.31
cg11405655		4.35E-05	0.022654	13.65	14.86	-1.21
cg27262054	ZFHX3	4.35E-05	0.022645	66.11	68.02	-1.91
cg17087741		4.37E-05	0.022666	68.62	72.73	-4.11
cg16071219	LPAR6	4.39E-05	0.022666	40.07	42.76	-2.69
cg06223834	ADCY9	4.40E-05	0.022666	76.29	80.08	-3.78

cg07683388	ANKRD44	4.40E-05	0.022666	58.24	60.24	-2.01
cg09357462	DUSP5	4.40E-05	0.022666	53.71	55.50	-1.80
cg22425466	RNF208	4.40E-05	0.022666	74.89	76.00	-1.11
cg23825480	MORC2	4.40E-05	0.022666	41.65	38.81	2.84
cg03384915	SIN3B	4.41E-05	0.022666	74.69	76.62	-1.94
cg07166654		4.41E-05	0.022666	3.62	3.08	0.54
cg00858599		4.42E-05	0.022666	9.85	10.42	-0.57
cg05985317	PRRT1	4.42E-05	0.022666	40.08	41.98	-1.90
cg07267166	ZNF323	4.42E-05	0.022666	6.54	6.99	-0.45
cg18900812	CDKN1A	4.43E-05	0.022666	14.92	16.21	-1.28
cg01414934	SH3BGRL3	4.45E-05	0.022714	7.99	8.42	-0.42
cg05329578		4.45E-05	0.022714	25.63	28.41	-2.78
cg21121843	HTT	4.46E-05	0.022717	22.25	24.23	-1.98
cg12103569	SFRS8	4.47E-05	0.022718	34.52	33.21	1.31
cg06968354	ZNF423	4.48E-05	0.022783	82.93	81.79	1.14
cg06394460	LNX2	4.51E-05	0.022902	49.73	52.69	-2.96
cg17873451	LOC440925	4.53E-05	0.022939	3.80	4.13	-0.33
cg26908328	SERINC5	4.53E-05	0.022945	9.38	10.24	-0.85
cg01955533	CDKN1A	4.57E-05	0.023083	19.63	20.37	-0.74
cg06065019		4.57E-05	0.023083	11.14	12.63	-1.49
cg13744172		4.58E-05	0.023083	12.44	13.13	-0.69
cg04359840	XYLT1	0.000046	0.023147	48.26	50.05	-1.79
cg05059607	PITPNC1	4.61E-05	0.023181	27.77	25.98	1.79
cg12689529	KIRREL3	4.67E-05	0.023481	6.80	7.40	-0.61
cg02101876		4.72E-05	0.023645	5.45	4.99	0.46
cg16702313	C14orf43	4.72E-05	0.023645	62.08	63.25	-1.17
cg08942527	EFNA5	4.74E-05	0.023741	77.25	75.90	1.35
cg24488469		4.75E-05	0.023771	15.10	16.03	-0.94
cg03547355		4.77E-05	0.02382	54.63	55.84	-1.21
cg14738823	ERP27	4.80E-05	0.023937	59.28	57.61	1.67
cg07877900	GPR114	4.81E-05	0.023948	9.60	8.81	0.79
cg07925587	KRT80	4.81E-05	0.023948	6.63	7.18	-0.55
cg08242636	CBFB	4.89E-05	0.024292	5.17	5.54	-0.37
cg24201362		4.89E-05	0.024284	87.94	89.79	-1.86
cg09162537	AP2A2	4.92E-05	0.024296	35.24	33.61	1.62
cg11666770		4.92E-05	0.024296	70.97	76.63	-5.65
cg26346875	VARS	4.92E-05	0.024296	5.79	6.16	-0.37
cg02740606	CORO1B	4.93E-05	0.024296	67.82	69.61	-1.79
cg22596049	GAS7	4.93E-05	0.024296	71.32	72.39	-1.07
cg26988138	GNG7	4.93E-05	0.024296	12.64	10.15	2.49
cg13299325		4.94E-05	0.024296	55.91	57.94	-2.03
cg12883279	PPT2	4.95E-05	0.024329	73.80	77.28	-3.49
cg10116432	NT5C2	4.96E-05	0.024329	23.22	24.65	-1.43
cg19202384	PYCR1	5.02E-05	0.024582	67.35	68.85	-1.50
cg26470501	BCL3	5.03E-05	0.024601	48.86	50.30	-1.44
cg24968629	CELSR1	5.04E-05	0.02463	69.57	65.77	3.80
cg24924577	SEMA4B	5.05E-05	0.02463	28.03	26.46	1.56
cg21879146		5.07E-05	0.024703	64.59	62.03	2.56
cg03233656	SLC1A4	5.09E-05	0.024787	23.77	25.93	-2.15

cg15930380	MIER1	5.11E-05	0.02486	26.41	24.50	1.91
cg19859980	C1orf97	5.15E-05	0.02503	2.65	2.83	-0.18
cg02573176	SLC10A5	5.17E-05	0.025077	30.09	28.71	1.38
cg16804020	KLF6	5.17E-05	0.025077	0.11	0.15	-0.04
cg24212517	GRN	5.21E-05	0.025186	9.07	9.59	-0.51
cg15554421	C3orf26	5.22E-05	0.025212	5.20	5.60	-0.39
cg01431482	PRDM16	5.23E-05	0.025212	53.27	55.07	-1.80
cg12033822	SLC35C2	5.25E-05	0.025304	25.89	26.89	-1.00
cg18515591	BTG4	5.27E-05	0.025362	8.02	8.54	-0.52
cg12421110	C13orf15	5.28E-05	0.025362	72.76	71.48	1.28
cg10807309	VARS	5.31E-05	0.025479	34.13	32.52	1.61
cg11261850		5.33E-05	0.025542	28.40	26.69	1.72
cg17256711	OPRM1	5.34E-05	0.025574	15.31	13.97	1.34
cg19406367	SGIP1	5.35E-05	0.025574	67.45	65.33	2.11
cg04519775		5.38E-05	0.025701	53.90	55.22	-1.31
cg09037630		5.39E-05	0.025737	10.61	11.42	-0.82
cg03427663		5.40E-05	0.025757	11.77	12.35	-0.58
cg04560741	LOC388796	5.41E-05	0.025773	79.14	80.01	-0.87
cg03294491	SMAD2	5.47E-05	0.025996	12.18	13.02	-0.84
cg07201017	FLJ41350	5.48E-05	0.026037	16.52	17.21	-0.69
cg12804677	PRDM16	5.49E-05	0.026075	65.48	66.78	-1.30
cg09710580	ZNF786	5.50E-05	0.026079	85.47	86.48	-1.01
cg24247537	PTDSS2	5.53E-05	0.026173	9.34	8.36	0.98
cg02968508	IL17RE	5.54E-05	0.026216	72.75	74.06	-1.31
cg10825315	TSHR	5.56E-05	0.026245	27.65	26.25	1.39
cg04119529	NTHL1	5.58E-05	0.026278	61.41	60.07	1.35
cg15030712	CHN2	5.58E-05	0.026278	19.95	20.89	-0.94
cg26790897	SUMF2	5.58E-05	0.026278	39.62	38.05	1.57
cg01911191		5.59E-05	0.026278	2.36	2.09	0.27
cg04425624	TNF	5.62E-05	0.026359	11.64	12.57	-0.93
cg07969918	PIK3IP1	5.62E-05	0.026357	30.45	31.54	-1.09
cg22159815	MIR29C	5.62E-05	0.026357	26.32	25.29	1.03
cg18946533	SH2D7	5.64E-05	0.026394	57.38	55.84	1.54
cg13087259		5.65E-05	0.026394	23.13	24.25	-1.12
cg06959021	TCHP	5.66E-05	0.026394	60.69	59.24	1.45
cg13934406	PPT2	5.66E-05	0.026394	77.85	80.60	-2.75
cg10894991		5.73E-05	0.026672	65.02	66.27	-1.25
cg23423933	CTTN	5.73E-05	0.026672	79.43	80.50	-1.07
cg01107874	C10orf41	5.74E-05	0.02668	77.93	79.73	-1.80
cg22526531	KIAA0319L	5.76E-05	0.026724	40.89	39.93	0.96
cg24095374	RGS14	5.76E-05	0.026724	5.37	5.79	-0.42
cg18369990	FBLN7	5.78E-05	0.026745	52.77	51.20	1.58
cg27514333	SMAD6	5.78E-05	0.026745	14.92	15.86	-0.93
cg09374353	EHD1	5.79E-05	0.026767	19.45	20.40	-0.94
cg03193328	TPST1	5.80E-05	0.026767	14.68	13.78	0.90
cg12864721	C10orf41	5.83E-05	0.02687	16.29	18.55	-2.26
cg16755922	FOXK2	5.83E-05	0.02687	65.14	62.81	2.33
cg26133399	FAM19A2	5.84E-05	0.026898	11.18	10.18	0.99
cg08006956	LSM4	5.85E-05	0.026915	65.98	67.35	-1.36

cg02512902	KSR1	5.87E-05	0.026968	59.81	61.32	-1.51
cg03061612	TTLL9	0.000059	0.027078	60.27	58.74	1.53
cg18033416	RHBDL3	5.92E-05	0.027112	58.63	60.37	-1.74
cg18335931	MAST3	5.94E-05	0.027161	19.14	20.38	-1.23
cg07756788		5.95E-05	0.027161	70.81	72.17	-1.36
cg09465703	JMJD8	5.95E-05	0.027161	11.40	12.93	-1.53
cg13096701	RAPGEF2	5.95E-05	0.027161	79.25	76.89	2.36
cg04843111	BCAN	5.98E-05	0.027247	50.87	52.60	-1.74
cg24173182	HIC1	6.09E-05	0.027736	31.98	29.56	2.42
cg09222732		6.16E-05	0.02801	82.22	83.50	-1.28
cg15114651	SLC1A5	6.18E-05	0.028027	49.22	50.50	-1.28
cg18691800	RBM24	6.18E-05	0.028027	0.34	0.27	0.08
cg04583842	BANP	6.20E-05	0.028052	35.19	33.23	1.96
cg16116321	FAM124B	6.20E-05	0.028052	50.17	51.89	-1.72
cg20698113	PIM3	6.20E-05	0.028052	75.49	76.78	-1.29
cg12029639	MIR548F5	6.22E-05	0.028078	26.77	28.49	-1.72
cg25753024	SSH3	6.22E-05	0.028078	3.14	3.65	-0.51
cg06501716	C22orf39	6.23E-05	0.028092	56.28	57.73	-1.44
cg22715788	IQCE	6.25E-05	0.028114	83.95	82.37	1.58
cg26878709	TMEM136	6.29E-05	0.028275	52.36	50.30	2.06
cg07298482	SUNC1	0.000063	0.028301	78.72	76.56	2.15
cg19220282	SLC1A4	6.31E-05	0.028301	22.02	24.99	-2.98
cg19319393	GPR153	6.31E-05	0.028301	24.00	22.66	1.34
cg24142464	PABPC4	6.37E-05	0.028529	10.27	10.95	-0.68
cg22950598	GDF11	6.38E-05	0.028545	61.81	63.40	-1.59
cg09636849		6.40E-05	0.028595	1.37	1.59	-0.21
cg25233339	ATP1B3	6.48E-05	0.028884	3.06	3.40	-0.34
cg07626482	SLC1A5	6.49E-05	0.028884	25.53	26.52	-0.99
cg17759095	DST	6.49E-05	0.028884	27.27	29.18	-1.91
cg05302701	NFATC1	6.51E-05	0.028969	79.23	80.39	-1.16
cg24852032	SYNGAP1	6.55E-05	0.029095	5.08	5.54	-0.46
cg08386696	BAHCC1	6.56E-05	0.02911	76.01	76.97	-0.95
cg03194226	CLEC3B	6.59E-05	0.029206	82.52	83.53	-1.01
cg04086928	RAPGEF1	6.59E-05	0.029206	64.15	65.68	-1.53
cg08606254	AHRR	6.60E-05	0.029216	84.70	83.12	1.58
cg13676312	DPT	6.63E-05	0.029312	78.59	79.53	-0.93
cg06060868	SDHA	6.64E-05	0.029312	75.45	76.52	-1.07
cg04997045	CTBP1	6.66E-05	0.02937	54.30	52.10	2.20
cg05871756		6.71E-05	0.029551	27.11	28.10	-1.00
cg00793186	NRG2	6.72E-05	0.029565	25.29	26.64	-1.35
cg02557933	SPEG	6.73E-05	0.029565	56.19	57.65	-1.46
cg04388657	RPL35	6.74E-05	0.029565	46.40	48.28	-1.88
cg07730673	PIGX	6.75E-05	0.029565	4.20	4.44	-0.24
cg08528970		6.75E-05	0.029565	14.31	14.91	-0.60
cg14950321	PLIN5	6.76E-05	0.029608	41.56	43.24	-1.68
cg11010122	DNAJB14	6.77E-05	0.02961	3.42	3.76	-0.34
cg00575674		6.79E-05	0.029642	20.07	21.88	-1.81
cg16481281	IL11	6.79E-05	0.029644	4.84	5.27	-0.43
cg01290345	TMEM104	6.83E-05	0.029769	6.71	7.16	-0.45

cg24242519	FAM49A	6.84E-05	0.029784	13.58	14.63	-1.05
cg04871173	MAD1L1	6.85E-05	0.029784	85.07	86.18	-1.11
cg25298754	ZBED2	6.86E-05	0.029796	49.09	50.61	-1.53
cg00308065	CCDC92	6.87E-05	0.029823	74.17	72.82	1.36
cg08062087	C2orf66	6.89E-05	0.029852	69.18	67.43	1.75
cg27574654	IL19	6.90E-05	0.029883	67.59	65.15	2.44
cg01005180	PRKAG2	6.95E-05	0.030012	54.13	55.43	-1.30
cg13092108	RPS6KA1	6.95E-05	0.030012	31.30	32.36	-1.06
cg24715767	PRDM2	6.95E-05	0.030012	59.88	58.10	1.78
cg13693328		6.96E-05	0.030012	25.23	26.34	-1.11
cg05626073	AP2A2	7.00E-05	0.030129	29.38	26.02	3.37
cg21503834	MAD1L1	7.00E-05	0.030129	77.70	76.37	1.33
cg07643930	ZNF598	7.01E-05	0.030129	8.77	9.40	-0.63
cg02135077		7.02E-05	0.030129	6.66	7.18	-0.53
cg06193043	NPPA	7.03E-05	0.030129	53.23	50.43	2.80
cg22396280		7.03E-05	0.030129	71.69	70.39	1.30
cg10520740	CACNA2D4	7.05E-05	0.030166	57.41	58.74	-1.32
cg13910681	FAM102A	7.05E-05	0.030166	10.04	10.62	-0.58
cg21884062	MIR548F5	7.08E-05	0.030262	4.18	4.53	-0.35
cg17971578	STK40	7.12E-05	0.03038	20.52	21.50	-0.98
cg01919999	GNB2L1	7.13E-05	0.03038	17.32	18.23	-0.91
cg11197664		7.14E-05	0.03038	71.62	68.68	2.94
cg08708790		7.15E-05	0.03038	58.51	59.58	-1.08
cg17755386	SNORD46	7.15E-05	0.03038	16.08	16.91	-0.84
cg10861146	EFNA1	7.16E-05	0.03038	13.18	13.94	-0.76
cg21190381	LOC100302	7.17E-05	0.030427	2.11	1.86	0.25
cg11701312	RPS5	7.23E-05	0.030628	48.45	49.77	-1.31
cg20306574	FITM1	7.26E-05	0.030675	60.02	57.64	2.37
cg00070899	GRM4	7.27E-05	0.030675	76.93	79.45	-2.52
cg11222173	RPTOR	7.27E-05	0.030675	61.29	59.24	2.05
cg21631918	STRA6	7.27E-05	0.030675	50.67	52.45	-1.78
cg06893362	C20orf199	7.28E-05	0.030675	58.29	59.90	-1.61
cg17133388	FAM162A	0.000073	0.030723	7.92	8.61	-0.70
cg09636313		7.37E-05	0.030986	59.78	57.65	2.13
cg10076395		7.37E-05	0.030986	71.31	69.85	1.46
ch.6.33611621F		0.000074	0.031047	1.72	1.94	-0.21
cg08397747	SPDYA	7.42E-05	0.031094	83.15	80.85	2.30
cg20317123	TCF21	7.43E-05	0.031094	12.69	13.35	-0.65
cg22011809	POPDC3	7.43E-05	0.031094	85.09	83.57	1.52
cg05898881		7.47E-05	0.031212	85.52	86.33	-0.82
cg07742396		0.000075	0.031329	41.90	43.34	-1.44
cg26280998		7.53E-05	0.03141	52.20	50.70	1.50
cg08324925	CSF2	7.55E-05	0.031417	60.21	61.53	-1.32
cg19190487	ITSN1	7.55E-05	0.031417	88.75	87.79	0.96
cg24524451	ADCK2	7.55E-05	0.031417	1.39	1.71	-0.32
cg20456732		7.58E-05	0.0315	66.44	67.93	-1.49
cg12559228	C19orf76	0.000076	0.031541	68.63	70.02	-1.39
cg27165835	PAOX	7.62E-05	0.031601	62.53	60.67	1.86
cg06518831	BAT4	7.63E-05	0.031601	11.00	11.58	-0.57

cg04039397	CD96	7.64E-05	0.031614	21.49	23.38	-1.89
cg08601457	FYN	7.69E-05	0.031781	6.42	7.02	-0.60
cg12621745	PLEC1	0.000077	0.031787	70.14	71.33	-1.19
cg15157628	C10orf128	0.000077	0.031787	86.14	86.94	-0.80
cg02672759		7.76E-05	0.03199	70.49	72.25	-1.75
cg05299486	ATP6V0A1	7.77E-05	0.032006	2.34	1.99	0.35
cg05238276		7.82E-05	0.032151	82.51	80.77	1.74
cg20388732	STAT5A	7.82E-05	0.032155	5.56	6.06	-0.51
cg01055579	NLE1	7.84E-05	0.032192	3.44	3.78	-0.34
cg27310311		7.88E-05	0.032327	79.48	80.42	-0.94
cg05548393	SLC30A8	0.000079	0.032353	78.63	76.65	1.98
cg06784339	MKRN1	7.97E-05	0.032606	5.22	5.57	-0.35
cg16718162	MDS2	8.03E-05	0.0328	9.37	10.04	-0.67
cg17333042	KAZALD1	8.08E-05	0.032976	37.69	38.97	-1.27
cg14780837	SYNGR1	8.12E-05	0.033094	10.09	10.95	-0.86
cg00082939	C10orf41	8.14E-05	0.033142	46.31	48.74	-2.43
cg06037693	RUNX3	8.15E-05	0.033149	76.20	77.40	-1.20
cg02149899		8.17E-05	0.033213	53.66	55.32	-1.66
cg14920846	NAV1	8.19E-05	0.033245	42.70	46.18	-3.48
cg15727507	B3GNTL1	8.19E-05	0.033245	45.79	44.30	1.49
cg07512814	LDLR	0.000082	0.033252	36.22	38.22	-2.00
cg08330132		8.22E-05	0.033268	80.25	81.73	-1.48
cg10012530	HS6ST1	8.23E-05	0.033275	70.75	72.12	-1.37
cg09182189	NADK	8.26E-05	0.033365	24.21	25.21	-1.00
cg07450086	SELT	8.27E-05	0.033365	22.91	24.07	-1.16
cg09006487	RYBP	8.27E-05	0.033365	18.17	19.36	-1.19
cg01677623	ARHGAP22	8.32E-05	0.033494	71.46	72.57	-1.12
cg25577463	VENTX	8.32E-05	0.033494	3.85	3.43	0.42
cg13594903	STOML2	8.34E-05	0.03353	7.06	7.70	-0.65
cg00295485	UXS1	8.37E-05	0.033584	43.03	44.73	-1.71
cg20780180	SNX24	8.37E-05	0.033584	59.96	61.74	-1.78
cg03378003	SETD2	8.38E-05	0.033584	85.00	84.01	0.99
cg16664394	CCNO	8.38E-05	0.033584	9.65	10.23	-0.57
cg01201215	ATOX1	0.000084	0.033585	59.03	60.51	-1.48
cg24771152	VARS	0.000084	0.033585	85.62	81.79	3.83
cg24531401	INTS1	8.42E-05	0.033627	21.67	22.51	-0.84
cg08305533	SFI1	8.44E-05	0.033663	41.73	40.06	1.67
cg17930194	LHX6	8.47E-05	0.03376	26.77	29.24	-2.46
cg08170227	ACTN1	8.48E-05	0.03376	43.25	45.03	-1.79
cg23063243	ONECUT1	8.49E-05	0.033779	10.22	10.92	-0.69
cg04726360		8.51E-05	0.033842	68.56	70.81	-2.25
cg17827670	AHCYL2	8.53E-05	0.03387	7.95	8.48	-0.54
cg08687386	IER3	8.58E-05	0.034032	4.88	5.18	-0.30
cg26853536	UBC	0.000086	0.034097	44.82	46.69	-1.87
cg01768201		8.72E-05	0.03452	4.51	4.84	-0.33
cg24560678		8.73E-05	0.034528	68.06	64.77	3.29
cg01081263	SCUBE2	8.84E-05	0.034931	33.69	35.34	-1.65
cg02055540		8.86E-05	0.034976	25.60	27.91	-2.30
cg05205351	NOP56	8.87E-05	0.034976	25.29	26.42	-1.13

cg18900669	CD68	8.88E-05	0.034976	13.79	14.56	-0.77
cg09931909	C6orf150	8.89E-05	0.035012	41.75	44.61	-2.86
cg22242842		8.96E-05	0.035222	8.22	9.13	-0.91
cg05256179	BAT5	8.99E-05	0.035309	83.24	84.26	-1.01
cg01744331	KCNQ1OT1	9.05E-05	0.035536	73.15	76.07	-2.92
cg06433467		9.11E-05	0.035713	6.23	7.05	-0.82
cg04806562	TEX19	9.12E-05	0.035736	38.24	37.14	1.10
cg02174884	TNNC2	9.25E-05	0.03618	50.97	52.69	-1.72
cg07063068	GPR68	9.25E-05	0.03618	4.59	5.20	-0.60
cg09570958	HS3ST3B1	9.28E-05	0.036244	14.60	15.58	-0.98
cg16143804	MSH5	9.31E-05	0.036324	6.41	6.77	-0.35
cg02104644	SYT7	9.34E-05	0.036386	19.45	20.90	-1.45
cg10047572	IER3	9.34E-05	0.036386	8.51	9.02	-0.51
cg21222350		9.38E-05	0.036516	66.52	67.71	-1.19
cg14560670	LOC145837	9.48E-05	0.036809	39.36	40.76	-1.41
cg25474070	IL3	9.49E-05	0.036827	64.30	65.67	-1.37
cg18327772	HMGB4	9.52E-05	0.03691	74.95	76.33	-1.38
cg14217495		9.53E-05	0.03691	8.21	8.97	-0.76
cg22977892		9.54E-05	0.036929	19.21	20.33	-1.13
cg26437697	SAMD4A	9.55E-05	0.036936	41.06	39.69	1.37
cg03784994	HCCA2	0.000096	0.037064	96.35	94.86	1.49
cg19696491	CHRNA5	0.000096	0.037064	44.40	42.22	2.18
cg14590011		9.62E-05	0.037091	34.85	36.25	-1.41
cg16379091		9.63E-05	0.037091	70.43	71.91	-1.48
cg04540406	SHCBP1	9.64E-05	0.037091	12.62	13.41	-0.79
cg19137107		9.66E-05	0.037135	45.06	46.80	-1.74
cg12827530	RNU5E	9.68E-05	0.037166	18.74	17.48	1.27
cg14614490	PHF19	0.000097	0.037224	60.55	61.71	-1.16
cg21268578	GGA1	0.000098	0.037539	6.04	6.61	-0.57
cg26879059		0.000098	0.037539	19.93	21.38	-1.46
cg24151027	SCAMP1	9.81E-05	0.037539	1.68	1.88	-0.20
cg06100161	RORA	9.83E-05	0.037589	63.20	65.14	-1.94
cg05593162	SDCCAG8	9.89E-05	0.037718	46.62	44.92	1.71
cg09142135	TCIRG1	9.89E-05	0.037718	83.10	84.39	-1.29
cg04531781		9.92E-05	0.037773	51.77	48.94	2.83
cg22162848	ITGB4	9.94E-05	0.037808	56.13	58.20	-2.07
cg12573705	MIR548F5	9.96E-05	0.037873	8.00	8.74	-0.74
cg06459104	EPB41L3	9.98E-05	0.037886	34.76	38.17	-3.41
cg26968812	TPM4	9.98E-05	0.037886	11.13	11.68	-0.55
cg05323272	TTC39A	9.99E-05	0.037886	80.07	84.02	-3.95
cg03514843	EMILIN1	0.0001	0.037907	61.91	63.42	-1.52
cg02895394	PRKDC	0.0001	0.037907	75.97	77.16	-1.18
cg00624799	ZNF710	0.0001	0.037972	75.36	76.84	-1.48
cg06177555	SPN	0.000101	0.03802	37.39	36.12	1.26
cg01286191	PRKDC	0.000101	0.038159	89.18	90.29	-1.11
cg13379236	EGF	0.000101	0.038159	62.37	60.91	1.47
cg04490079	OR5K2	0.000101	0.038159	79.42	77.18	2.25
cg26890706	RNF182	0.000101	0.038159	61.89	64.04	-2.15
cg13278353		0.000101	0.038159	13.61	14.92	-1.31

cg11069824	CHD5	0.000102	0.03823	56.74	58.15	-1.41
cg00469602		0.000102	0.038311	17.04	18.04	-1.00
cg22490254		0.000102	0.038311	15.21	13.94	1.27
cg08287903	UGT8	0.000103	0.038472	50.70	48.90	1.80
cg20550262		0.000103	0.038529	75.44	77.80	-2.36
cg02218496		0.000103	0.038529	26.85	27.92	-1.07
cg02998240		0.000103	0.038529	61.61	64.61	-3.00
cg20530056	IKBKE	0.000103	0.038575	65.58	66.87	-1.29
ch.1.159013533R		0.000103	0.038575	3.68	3.92	-0.24
cg18526008		0.000104	0.038701	4.34	4.68	-0.34
cg09425279		0.000104	0.038701	62.76	61.35	1.41
cg18507732		0.000104	0.038701	52.49	54.34	-1.84
cg22686881	TLX3	0.000105	0.038895	11.51	10.76	0.75
cg13359998	GALNT2	0.000105	0.038973	13.61	14.57	-0.96
cg17993335	DNMBP	0.000106	0.039177	78.67	76.86	1.81
cg16492833	C6orf125	0.000106	0.039177	9.74	10.67	-0.94
cg12044210	APBA2	0.000106	0.039177	70.06	68.94	1.12
cg18350524	UBE2N	0.000106	0.039177	8.08	8.70	-0.63
cg02013841		0.000106	0.039177	59.55	61.88	-2.34
cg08259514		0.000106	0.0393	4.14	3.61	0.53
cg02470690		0.000107	0.039306	8.27	8.82	-0.56
cg16151960	PHF15	0.000107	0.039577	60.22	61.39	-1.17
cg01668352	SRGAP1	0.000108	0.039665	75.92	77.17	-1.25
cg00013899	CACHD1	0.000108	0.039679	74.84	72.95	1.89
cg10317175		0.000108	0.039731	29.80	30.91	-1.11
cg25642234	PLBD1	0.000108	0.03979	57.82	59.64	-1.82
cg08173709	FOXP1	0.000109	0.039871	48.18	46.96	1.22
cg06695691	SPATA5	0.00011	0.040149	49.68	47.12	2.56
cg13816423	FOXP4	0.00011	0.040216	49.88	51.64	-1.76
cg19265289	ARRB2	0.00011	0.040336	5.18	5.54	-0.36
cg21010646	TTC24	0.00011	0.040345	75.78	77.40	-1.62
cg12306833		0.000111	0.040603	74.44	75.54	-1.11
cg14958080	TERT	0.000112	0.040783	89.24	90.14	-0.89
cg04992852		0.000112	0.040799	85.31	83.87	1.44
cg07949433	SCARNA16	0.000112	0.040799	8.10	8.66	-0.56
cg14588638	EWSR1	0.000112	0.040799	5.47	5.84	-0.37
cg06285727	ATG16L2	0.000112	0.040801	8.29	8.91	-0.62
cg02010481	JAZF1	0.000112	0.040806	11.73	12.63	-0.90
cg18295744	ZMIZ1	0.000113	0.040948	64.00	65.10	-1.09
cg11152412	EDC3	0.000114	0.041351	8.96	9.53	-0.57
cg25212025	PARD3	0.000114	0.041351	32.33	33.96	-1.63
cg26840970	ZNF19	0.000114	0.041351	70.89	69.03	1.86
cg13066481	MYLK	0.000114	0.041416	41.75	42.94	-1.19
cg02104700	S100P	0.000115	0.041492	2.56	2.76	-0.19
cg26722342	ITGA2	0.000115	0.041555	67.89	69.60	-1.71
cg02962647		0.000115	0.041555	64.24	62.87	1.37
cg02797271	GPR132	0.000116	0.041752	6.83	7.30	-0.46
cg16312514	SHANK2	0.000116	0.041787	16.53	15.25	1.29
cg14093936	SEMA7A	0.000117	0.041937	28.11	28.99	-0.88

cg00631221	REPS1	0.000117	0.041976	7.83	8.43	-0.60
cg13698937	C4orf46	0.000117	0.04202	77.10	75.15	1.95
cg03594515		0.000117	0.04202	9.77	10.28	-0.51
cg17983064	IL3	0.000117	0.04202	45.47	43.68	1.79
cg26457878	ZNF815	0.000118	0.042092	5.45	4.93	0.52
cg08867399	HS6ST1	0.000118	0.042092	57.15	59.67	-2.52
cg08519905	CD9	0.000118	0.042092	26.49	27.33	-0.84
ch.4.13872363R		0.000118	0.042267	0.53	0.41	0.11
cg19034132		0.000119	0.042473	3.65	3.95	-0.30
cg15378486		0.000119	0.042549	33.99	35.14	-1.15
cg09250678		0.00012	0.042603	6.86	7.75	-0.89
cg17662387	ABLIM1	0.00012	0.042617	3.50	2.88	0.62
cg09166091		0.00012	0.042679	15.34	14.46	0.88
cg15815084	DGKE	0.00012	0.042714	47.83	51.10	-3.27
cg10801607	SLC3A1	0.00012	0.042772	18.16	17.29	0.87
cg22006825	HNRNPUL1	0.000121	0.042772	19.93	20.73	-0.80
cg05665093	OBFC2A	0.000121	0.042905	82.76	80.92	1.84
cg21310090	PLTP	0.000121	0.042929	33.90	33.05	0.85
cg09372617	NEK7	0.000121	0.042929	60.66	58.74	1.92
cg01836137	INF2	0.000122	0.043184	47.14	45.52	1.62
cg00099441		0.000122	0.043184	6.23	6.81	-0.58
cg19028369	C3orf19	0.000123	0.043252	4.47	4.72	-0.25
cg02646480		0.000123	0.043263	80.73	81.82	-1.09
cg06491451	HSBP1L1	0.000123	0.043263	59.92	61.41	-1.49
cg27569706	LTBP1	0.000123	0.043458	27.96	29.53	-1.57
cg22501483		0.000124	0.043637	72.04	70.94	1.10
cg14621336		0.000124	0.043637	76.91	78.11	-1.20
cg14242895	MLL	0.000125	0.043734	57.22	55.74	1.48
cg03509898	ZFYVE21	0.000125	0.043858	78.42	77.02	1.40
cg06397161	SYNGR1	0.000125	0.043895	44.19	45.71	-1.52
cg11404945		0.000125	0.043903	75.93	77.08	-1.15
cg00300750	OSR2	0.000125	0.043903	4.14	4.40	-0.26
cg04985185	MBTPS1	0.000126	0.044232	84.52	82.91	1.61
cg20926024		0.000127	0.04424	8.48	8.97	-0.49
cg16872613		0.000127	0.044337	71.96	70.63	1.33
cg05289698	GCNT3	0.000127	0.044356	69.62	67.80	1.82
cg05277504	ASPSCR1	0.000128	0.044519	90.01	91.31	-1.30
cg14610746	CLN6	0.000128	0.0446	3.10	3.34	-0.24
cg13428066	KCNQ1	0.000128	0.0446	33.20	31.97	1.23
cg10270430	GRM4	0.000128	0.0446	66.35	68.51	-2.16
cg01609214	MIR30D	0.000129	0.044645	90.75	88.27	2.49
cg00246451	ARHGEF2	0.000129	0.044645	5.03	5.50	-0.48
cg08278008		0.000129	0.044715	6.37	6.94	-0.56
cg23680451	KLF6	0.000129	0.044778	13.91	15.11	-1.20
cg03505125	TRIM75	0.000129	0.044778	73.37	74.46	-1.09
cg24271203	IL31	0.000129	0.044778	65.26	66.65	-1.39
cg27366280	TRAK1	0.00013	0.044798	71.00	72.16	-1.17
cg08354053	RHBDL3	0.00013	0.044798	55.18	56.54	-1.36
cg19399165	EMILIN1	0.00013	0.045004	59.99	62.11	-2.12

cg20938359	SLC6A12	0.000131	0.045046	26.71	25.77	0.93
cg02571448	PCBP3	0.000131	0.045046	42.58	44.30	-1.72
cg00063111	SNORA6	0.000131	0.045046	13.69	15.11	-1.42
cg05525812		0.000131	0.045136	15.87	16.79	-0.91
cg11363234		0.000131	0.045136	62.70	64.41	-1.71
cg15723874	MEF2D	0.000132	0.045136	65.74	66.97	-1.23
cg22079019	ECEL1	0.000132	0.045136	66.66	68.20	-1.54
cg14179401		0.000132	0.045228	34.93	35.88	-0.94
cg22112435	POMGNT1	0.000132	0.045304	5.41	5.91	-0.50
cg23928726	PEX10	0.000133	0.045312	25.12	26.57	-1.45
cg26682866	CDK5R2	0.000133	0.045312	4.78	5.04	-0.26
cg24980413	AHRR	0.000133	0.045312	61.41	58.99	2.42
cg23670519		0.000133	0.045312	26.23	23.99	2.24
cg18490846	C17orf73	0.000133	0.045409	65.31	66.45	-1.14
cg18033092	C11orf49	0.000133	0.045409	50.13	48.92	1.21
cg27430293		0.000133	0.045409	36.27	34.29	1.99
cg15058557	SYNC	0.000134	0.045447	67.24	68.47	-1.23
cg17472111		0.000134	0.045496	21.65	20.84	0.81
cg01357381		0.000134	0.045496	7.38	7.75	-0.37
cg22114489		0.000134	0.045517	12.79	10.97	1.81
cg25722983	STK40	0.000134	0.045517	46.98	48.30	-1.33
cg12076915		0.000134	0.045517	61.35	63.31	-1.96
cg10898310	UBAC2	0.000135	0.045604	53.56	52.22	1.34
cg13705391	PRRT1	0.000135	0.045634	19.36	21.00	-1.64
cg08778287	IGF1R	0.000135	0.045634	32.47	30.40	2.07
cg09811127	MORN1	0.000135	0.045634	80.57	81.41	-0.83
cg13595518	DAB2IP	0.000135	0.045634	1.22	1.45	-0.22
cg22892904	CBX2	0.000135	0.045634	32.38	33.52	-1.14
cg27418217	HOMER2	0.000136	0.045661	22.82	22.04	0.78
cg17493193	PDIK1L	0.000137	0.045927	81.60	80.56	1.04
cg12279125		0.000137	0.045954	70.57	68.54	2.03
ch.8.49582343R		0.000137	0.045954	1.94	2.11	-0.17
cg20889322		0.000137	0.046028	15.73	16.89	-1.16
cg24541193	SLC19A2	0.000138	0.046129	51.04	48.91	2.14
cg18745507	ZGLP1	0.000138	0.046149	57.12	55.77	1.35
cg00255726	DOK2	0.000138	0.046326	7.54	6.95	0.59
cg27631057		0.000139	0.046381	71.59	73.48	-1.89
cg16365445	ITGB1	0.000139	0.046392	82.87	81.50	1.37
cg10612237	ZMIZ1	0.000139	0.046392	5.14	5.49	-0.35
cg03867607	MYL6	0.000139	0.046392	4.54	4.93	-0.39
cg10232140	ERCC6	0.000139	0.046392	1.74	1.40	0.34
cg10691866	TPST1	0.00014	0.046487	37.68	39.88	-2.20
cg13734518	DLD	0.00014	0.046556	85.85	84.15	1.70
cg04811945	CD38	0.00014	0.046556	14.48	13.70	0.79
cg26196424	C14orf37	0.00014	0.046625	57.26	59.10	-1.84
cg22190721	ESRP2	0.000141	0.04663	79.27	80.78	-1.51
cg02325250	CSF2	0.000141	0.04663	48.96	50.30	-1.34
cg05208153	C9orf25	0.000141	0.046649	75.78	76.81	-1.04
cg13800721	PRDM16	0.000141	0.04668	75.42	76.40	-0.98

cg21381845	PRKAR1B	0.000141	0.046704	53.15	54.58	-1.43
cg14521421	ST5	0.000141	0.04673	74.67	75.85	-1.17
cg05637113	SRFBP1	0.000141	0.04673	69.60	68.24	1.37
cg05047401	SEMA4A	0.000143	0.047225	8.96	7.92	1.04
cg03220447	NAV2	0.000143	0.047225	12.77	13.38	-0.61
cg09068286	GFPT2	0.000144	0.047225	51.20	53.27	-2.07
cg07285276	RAPGEF1	0.000144	0.047225	72.23	73.53	-1.31
cg18473686	SYN3	0.000144	0.047225	47.29	48.79	-1.50
cg16565913	RBM47	0.000144	0.047225	7.35	6.59	0.77
cg25115829	SUZ12P	0.000144	0.047225	74.42	72.26	2.15
cg01970407	AHRR	0.000144	0.047225	76.38	74.53	1.85
cg17831440	MCF2L	0.000144	0.047225	83.53	81.56	1.97
cg13722127	RARRES2	0.000144	0.047225	23.78	25.17	-1.39
cg04890576		0.000144	0.047225	26.60	24.18	2.42
cg24512005	NAP1L4	0,000144	0,047225	23,09	24,00	-0,91
cg19048950	LOC100188	0,000144	0,047238	73,63	71,88	1,75
cg00216361	GAP43	0,000145	0,047328	3,53	3,74	-0,22
cg05894970	CDGAP	0,000145	0,047328	14,62	15,48	-0,86
cg16022904	RPS6KA1	0,000145	0,047328	23,14	23,89	-0,75
cg13989999	BCL2L1	0,000145	0,047328	48,29	49,67	-1,38
cg14774585	NBEA	0,000145	0,047328	23,53	25,23	-1,70
cg24482246		0,000146	0,047372	81,44	79,98	1,46
cg23639734	SOD3	0,000146	0,047372	71,37	70,27	1,09
cg00123478	ZNF518B	0,000146	0,047419	41,60	43,28	-1,69
cg16844292	NCRNA0017	0,000146	0,047419	82,16	83,10	-0,95
cg22805381		0,000146	0,047456	55,78	57,42	-1,64
cg10454258	IL21R	0,000147	0,047504	31,35	30,31	1,05
cg17241657	AP1AR	0,000147	0,047551	1,53	1,39	0,15
cg19744173	FBLN7	0,000147	0,047716	17,11	16,32	0,79
cg04324758	KLF2	0,000148	0,047721	2,28	2,57	-0,29
cg03884592	HIVEP3	0,000148	0,047743	25,92	23,16	2,76
cg08742502	PRDM1	0,000148	0,047794	42,06	44,28	-2,22
cg17676574	UROC1	0,000148	0,04782	69,51	70,59	-1,08
cg17496794	PRKDC	0,000149	0,047831	86,68	87,63	-0,95
cg09205438	GABRG3	0,000149	0,047957	59,49	57,98	1,51
cg04195000	TTC38	0,000149	0,048021	79,24	78,18	1,07
cg14254419	AMPD2	0,00015	0,048261	3,14	3,31	-0,17
cg00313914	NAV1	0,000151	0,048603	43,80	48,37	-4,57
cg10665960	EPC2	0,000152	0,048831	4,78	5,04	-0,27
cg00872580	PFKFB3	0,000153	0,048929	67,39	66,11	1,28
cg12616487	ROM1	0,000153	0,048955	43,68	44,80	-1,12
cg02890250	HSH2D	0,000153	0,048955	79,45	77,85	1,60
cg21005412	WDR43	0,000153	0,048999	38,67	37,73	0,94
cg07620039		0,000154	0,0491	4,96	5,29	-0,33
cg04987734	CDC42BPB	0,000154	0,0491	32,49	31,05	1,44
cg17532753	HDAC4	0,000154	0,0491	68,68	66,81	1,87
cg16341979	IFI27	0,000155	0,04945	78,77	79,81	-1,04
cg06321596	XYLT1	0,000156	0,049619	27,35	28,94	-1,59
cg22934035	LTB	0,000156	0,049662	3,38	3,63	-0,24

cg07411111	TPD52L2	0,000156	0,049724	62,69	60,75	1,94
cg00041047	SLC6A16	0,000157	0,049724	81,20	77,76	3,45
cg09893465	INPP5A	0,000157	0,049724	87,32	83,93	3,39
cg05789250	C6orf48	0,000157	0,049726	18,35	19,98	-1,63
cg16515974	DDAH2	0,000157	0,049733	73,72	74,70	-0,98
cg06627354	TRPM8	0,000158	0,049889	63,78	61,77	2,00

Suppl. Table S3: Overlap between the signals significant ($p<0.05$) for sex-specific rank differences in each resampling cycle and those observed in the full population ("target list", shown in Tables 2 and 3). The cumulative mean overlap for a given resampling round is calculated as the average of the % overlaps of the current and all preceding resamplings.

resampling round	epigenetics (target N=92)			transcriptomics (target N=26)		
	overlap			overlap		
	overlap with target list (N)	% overlap	cumulative mean overlap (%)	overlap with target list (N)	% overlap	cumulative mean overlap (%)
1	28	30.4	30.4	17	68.0	68.0
2	47	51.1	40.8	15	60.0	64.0
3	53	57.6	46.4	15	60.0	62.7
4	50	54.3	48.4	12	48.0	59.0
5	46	50.0	48.7	13	52.0	57.6
6	49	53.3	49.5	6	24.0	52.0
7	48	52.2	49.8	14	56.0	52.6
8	48	52.2	50.1	13	52.0	52.5
9	51	55.4	50.7	7	28.0	49.8
10	43	46.7	50.3	15	60.0	50.8

Suppl. Fig. 1: Distribution of p-values of sex-specific rank differences of transcriptomic (N=350; left) and epigenetic (N=1,273; right) signals. The dotted lines indicate the limit of p=0.05 and the blue bars the corresponding signals.

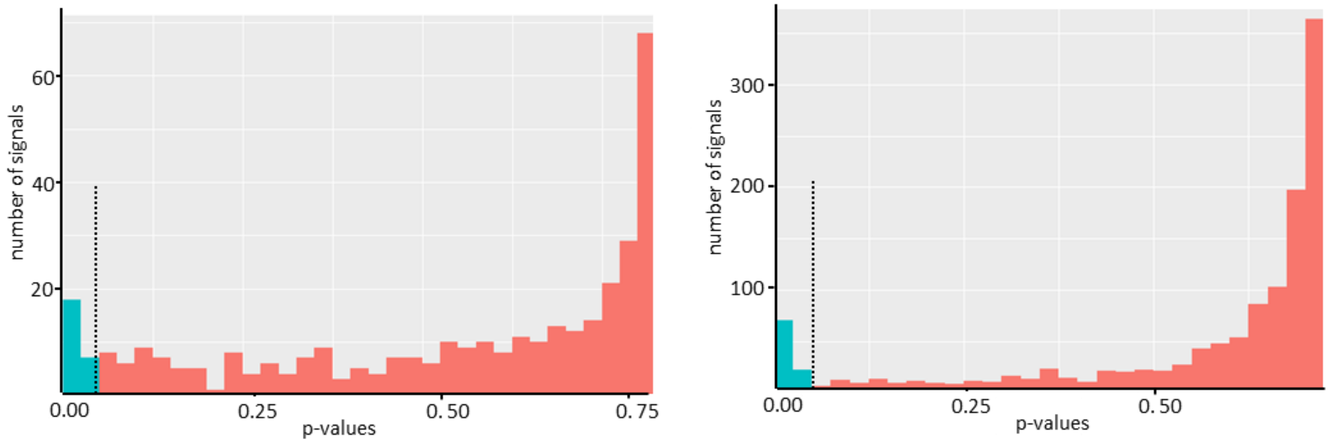

Suppl. Fig. S2: Cumulative overlap with target signals during 10 resamplings (arranged in random order) with sex-balanced populations; each y-axis value represents the average overlap of the corresponding resampling round and all preceding ones.

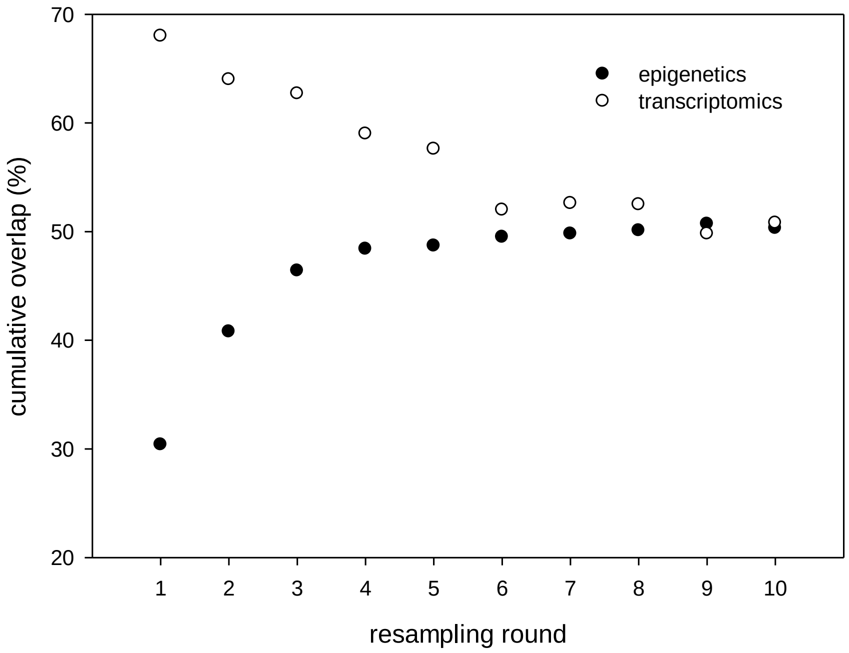

Supplement: Supplementary Information [file srep42870-s1.pdf]
